# RNA Sequencing Unveils Very Small RNAs With Potential Regulatory Functions in Bacteria

**DOI:** 10.3389/fmolb.2022.914991

**Published:** 2022-06-03

**Authors:** Idrissa Diallo, Jeffrey Ho, David Lalaouna, Eric Massé, Patrick Provost

**Affiliations:** ^1^ CHU de Québec Research Center/CHUL Pavilion, Department of Microbiology, Infectious Diseases and Immunology, Faculty of Medicine, Université Laval, Quebec City, QC, Canada; ^2^ CRCHUS, RNA Group, Department of Biochemistry and Functional Genomics, Faculty of Medicine and Health Sciences, Université de Sherbrooke, Sherbrooke, QC, Canada

**Keywords:** RNA sequencing, bacteria, *E. coli*, outer membrane vesicle (OMV), very small RNA (vsRNA), tRNA fragment (tRF)

## Abstract

RNA sequencing (RNA-seq) is the gold standard for the discovery of small non-coding RNAs. Following a long-standing approach, reads shorter than 16 nucleotides (nt) are removed from the small RNA sequencing libraries or datasets. The serendipitous discovery of an eukaryotic 12 nt-long RNA species capable of modulating the microRNA from which they derive prompted us to challenge this dogma and, by expanding the window of RNA sizes down to 8 nt, to confirm the existence of functional very small RNAs (vsRNAs <16 nt). Here we report the detailed profiling of vsRNAs in *Escherichia coli*, *E. coli*-derived outer membrane vesicles (OMVs) and five other bacterial strains (*Pseudomonas aeruginosa* PA7, *P. aeruginosa* PAO1, *Salmonella enterica* serovar Typhimurium 14028S, *Legionella pneumophila* JR32 Philadelphia-1 and *Staphylococcus aureus* HG001). vsRNAs of 8–15 nt in length [RNAs (8-15 nt)] were found to be more abundant than RNAs of 16–30 nt in length [RNAs (16–30 nt)]. vsRNA biotypes were distinct and varied within and across bacterial species and accounted for one third of reads identified in the 8–30 nt window. The tRNA-derived fragments (tRFs) have appeared as a major biotype among the vsRNAs, notably Ile-tRF and Ala-tRF, and were selectively loaded in OMVs. tRF-derived vsRNAs appear to be thermodynamically stable with at least 2 G-C basepairs and stem-loop structure. The analyzed tRF-derived vsRNAs are predicted to target several human host mRNAs with diverse functions. Bacterial vsRNAs and OMV-derived vsRNAs could be novel players likely modulating the intricate relationship between pathogens and their hosts.

## 1 Introduction

Largely superior than low-resolution techniques (Sanger sequencing, tiling microarrays, etc.), which do not provide a global view of the transcriptome (Barquist and Vogel, 2015), RNA sequencing (RNA-seq) technologies have been instrumental in elucidating the small non-coding RNA (ncRNA) profile of bacteria. The first small regulatory ncRNA (sRNA) micF that controls gene expression (Mizuno et al., 1984; Andersen et al., 1987), was discovered and characterized long before the well-known microRNAs (miRNAs) (Lee et al., 1993). The whos, whats, wheres, and whens of bacterial sRNAs have been widely discussed in noteworthy literature reviews (Wagner and Romby, 2015; Carrier et al., 2018; Jørgensen et al., 2020). They are diverse (cis or trans-encoded base pairing sRNAs, riboswitches, protein activity modulator, CRISPR sRNA, etc.) and act as through a variety of mechanisms (Waters and Storz, 2009).

sRNAs do not originate solely from intergenic regions (IGRs) (Argaman et al., 2001). Numerous studies reported the existence of many alternate genomic reservoirs of sRNAs (Chao et al., 2012; Guo et al., 2014; Chao and Vogel, 2016; Jose et al., 2019). Studies performed mainly in eukaryotes have shown that transfer RNAs (tRNAs) are an emerging source of functional sRNAs in the tRNA-derived fragments (tRFs) they generate (Kumar et al., 2016; Kuscu et al., 2018). Eukaryotic tRFs are involved in several functions ranging from translation inhibition to repression (Keam and Hutvagner, 2015; Kazimierczyk et al., 2022; Liu et al., 2022). In bacteria, little is known about the biogenesis, characteristics and cell-autonomous function of tRFs [for reviews, see ref. (Lalaouna et al., 2015; Li and Stanton, 2021)]. Furthermore, their potential contribution to host-bacteria interactions mediated by extracellular vesicles, such as outer membrane vesicles (OMVs), remain poorly documented.

The Gram-negative bacterial OMVs are first and foremost an efficient secretion and delivery system which contain various biologically active molecules such as outer-membrane proteins, lipopolysaccharide (LPS), periplasmic and cytoplasmic proteins as well as nucleic acids (DNA and RNA) (Yu et al., 2018). The OMVs have essentially three functions, including bacterial chances for survival (stress response, nutrient acquisition), regulation of microbial interactions within bacterial communities and promotion of pathogenesis (virulence factors delivery, modulation the host immune system modulation) (Kulp and Kuehn, 2010).

The standardization of sequencing allowed further exploration of bacterial extracellular RNAs, especially those potentially encapsulated in OMVs, which are known, among other intracellular functions, to regulate microbial interactions (bacteria-bacteria; host-bacteria) and facilitate pathogenesis [for review, see ref. (Ellis and Kuehn, 2010; Schwechheimer and Kuehn, 2015)]. Ghosal et al. (2015) realized one of the first in-depth characterizations of *E. coli* RNA contained in OMVs. They found that the majority of the sequenced reads are shorter than 40 nucleotides (nt) in length and mainly derive from tRNAs (tRFs).

Profile analysis of uropathogenic *E. coli* (Blenkiron et al., 2016) (UPEC, 536), *Porphyromonas gingivalis* (Ho et al., 2015), and *Vibrio cholerae* (O1 El Tor) (Sjöström et al., 2015) OMVs confirmed the presence of a wide variety of RNA biotypes and demonstrated that they can be internalized by human cells. The OMV-derived RNAs may play an active role in the host-bacteria interaction, as demonstrated for periodontal pathogens (Choi et al., 2017) and a 24-nt Met-tRF contained in OMVs of *P. aeruginosa* (Koeppen et al., 2016). These findings support the existence of a universal mechanism for intercellular communication mediated by bacterial OMV-derived RNAs that is conserved among bacterial species (Tsatsaronis et al., 2018; Li and Stanton, 2021). We recently discussed the possible role of bacterial sRNAs and their emergence as virulence factors in host-pathogen interactions, notably through OMVs (Diallo and Provost, 2020).

Under the premise that they are degradation products or too small to have biological functions or relevance, RNAs shorter than 16 nucleotides (nt) are systematically excluded from small RNA library construction, bioinformatics analyses and datasets (Blenkiron et al., 2016; Choi et al., 2017; Lambert et al., 2019). Nevertheless, a technical irregularity led to the serendipitous discovery of an RNA species of 12 nt corresponding to the 5′ half of let-7 microRNA, and coined semi-miRNA (smiRNA). Using a reporter gene activity assay in cultured human cells, we found that a smiRNA could modulate the gene regulatory effects of the miRNA from which it derived (Plante et al., 2012). Further RNA-seq analyses confirmed the existence of abundant and functional 12 and 13-nt dodecaRNAs (doRNAs) derived from 5.8S ribosomal RNA (rRNA) (Lambert et al., 2021), prompting us to inquire whether bacteria and their OMVs also contain unusually short RNAs.

In the present study, we used small RNA-seq to analyze the sRNA profile of *Escherichia coli* and their OMVs in the 8 to 30-nt RNA size window, together with five other bacterial strains, including 4 gram-negative (*P. aeruginosa* PAO1 and PA7, *L. pneumophila* JR32 Philadelphia-1, *S. Typhimurium* 14028S) and one gram-positive strain (*S*. *aureus* HG001). We report that bacteria and their OMVs contain large amounts of heterogenous very small RNAs (vsRNAs) shorter than 16 nt. Our results support the presence of thermodynamically stable and potentially functional tRFs, and the loading of selective vsRNAs in *E. coli* OMVs.

## 2 Materials and Methods

### 2.1 Bacterial Strains and Culture Condition

Bacterial strains and growth conditions used in this study are indicated in Supplementary Table S1. Unless otherwise stated, the reference strain of the study is *E. coli* K12 MG1655. Bacteria were grown at 30, 37, or 44°C on solid Luria-Bertani (LB) agar plates, in liquid LB medium, in AYE or in BHI media at 250 RPM.

### 2.2 Outer Membrane Vesicles Isolation and Characterization

#### 2.2.1 Outer Membrane Vesicles Isolation

OMVs were isolated from the reference *E. coli* strain K-12 MG1655 as described previously with modifications (Chutkan et al., 2013; Klimentová and Stulík, 2015) or with the Exobacteria™ Kit (System Biosciences, CA, United States, Cat. No. EXOBAC100A-1) following the manufacturer’s instructions.

Briefly, *E. coli* cells were grown in 120 ml of LB (in 1 L flask) to an OD_600nm_ of ∼0.5, upon which bacteria were pelleted by centrifugation at 10,000 × g for 10 min at 4°C and discarded. The supernatant was then successively filtered through a 0.45 and 0.2 µm-pore size VacuCap™ filters (PALL, MI, United States, Cat No. 4634, TA4632), and an inoculum was taken to confirm the absence of bacteria on LB plates. Thereafter, OMVs were pelleted by ultracentrifugation and washed twice with PBS at ∼200,000 × g for 2 h at 4°C in a Thermo Scientific Sorvall™ WX+ Ultracentrifuge equipped with the T-1250 Fixed Angle Rotor. After removing the supernatant, OMVs were resuspended in 200 μl sterile PBS.

Isolation of OMVs by the Exobacteria™ Kit uses precipitation-free gravity column system. Based on the same approach, 30 ml of pre-cleared (centrifuged and filtered as above) bacterial culture were used to harvest OMVs following the manufacturer’s instructions. Using this method, bacterial OMVs were eluted with 1.5 ml of elution buffer.

Purified OMVs were subjected to quality control (QC) and characterization, including LB plating to ensure lack of bacterial contamination, Coomassie blue-stained protein gel, transmission electron micrograph (TEM), and dynamic light scattering (Zetasizer). The pellet was stored at −80°C before being used for downstream applications. Complementary experiments consisting of RNase A treatment of OMVs were performed to verify the interiorization of their sRNAs content.

#### 2.2.2 Coomassie Blue Staining

Following dilution in 6X SDS protein loading buffer, 5 µg of OMVs (protein content), total protein extracts from *E. coli* MG1655 and fractions of intermediate stages (supernatant filtrate 0.45 or 0.22 µm) were charged on a 7.5% Mini-PROTEAN^®^ TGX™ Precast Protein Gel (BIORAD, Cat No. 4561025) with the recommended running buffer (Tris-Glycine buffer). The gel was then soaked for 5 min with shaking in 0.5% Coomassie Blue G-250 (Thermofisher, Cat. No. 20279; prepared in 50% methanol, 10% acetic acid). The excess staining was removed with the destaining solution (40% methanol and 10% acetic acid) to allow visualization of proteins as blue bands on a clear background.

#### 2.2.3 Electron Microscopy

Different OMV dilutions were analyzed by the Microscopy Platform of the Institut de biologie intégrative et des systèmes, Université Laval, Quebec City, QC, Canada. OMVs were treated as described previously (Benmoussa et al., 2017) and observed using the JEOL^®^ electron microscope 1230 (JEOL^®^ Ltd. Akishima, Tokyo) operating at an acceleration voltage of 80 kV.

#### 2.2.4 Particle Size Measurements

A Zetasizer Nano-ZS (Malvern Ltd.) light-scattering measurement system was used to determine the hydrodynamic size of OMVs. 100 µl of different OMV samples were loaded in a UV cuvette micro (BRAND), and the particle size was measured (three averaged measurements) at 4°C.

### 2.3 RNA Analysis

#### 2.3.1 RNA Isolation

Total RNA from bacterial strains was extracted using the hot phenol procedure (Aiba et al., 1981) or the RiboPure™ Bacteria Kit (Invitrogen, ON, Canada, Cat. No. AM 1925) following the manufacturer’s recommendations. OMV-derived RNAs were isolated with RiboPure™ Bacteria Kit or RNAzol RT (Sigma, MO, United States, Cat. No. R4533) reagents following the manufacturer’s recommendations. RNAzol RT kit allowed selective isolation (enrichment) of small RNAs (<200 nt). All RNA samples were subjected to treatment with DNase-I when applicable, quantified with the NanoDrop™ 2000 Spectrophotometer (Thermo Scientific™, Cat. No. ND-2000) and saved at −80°C before downstream applications.

#### 2.3.2 Illumina Nextseq Sequencing

##### 2.3.2.1 Experiment Workflow

The experimental workflow and the flowchart of data analysis are detailed in Supplementary File SA.

##### 2.3.2.2 RNA Sample Quality Control

RNA concentration was determined by OD_260_ using a NanoDrop ND-1000 instrument, and the OD_260_/OD_280_ ratio calculated for quality control. RNA integrity and gDNA contamination were tested by denaturing agarose gel electrophoresis. The results are summarized in Supplementary File SB.

##### 2.3.2.3 Illumina HiSeq Sequencing

For each biological condition, an RNA sample was prepared by pooling equivalent amounts of total RNA isolated from 2 or 3 biological replicate samples. In order to obtain the most representative data, we opted for a pooling strategy so to minimize the influence of interindividual variability. Previous studies have shown it to be a valid alternative to biological replicates at much reduced cost for large-scale gene expression approaches (Kendziorski et al., 2003; Kendziorski et al., 2005; Glass et al., 2005).

Total RNA was shipped on dry ice to the sequencing platform of Arraystar Inc. (Rockville, MD, United States). Total RNA of each sample was used to prepare the sRNA sequencing library which included the following steps: 1) 3′ adapter ligation with T4 RNA ligase 2 (truncated); 2) 5′ adapter ligation with T4 RNA ligase; 3) cDNA synthesis with RT primer; 4) PCR amplification; 5) extraction and purification of ∼130–150 bp PCR amplified fragments from the PAGE gel. After the completed libraries were quantified with Agilent 2,100 Bioanalyzer, the DNA fragments in the libraries were denatured with 0.1 M NaOH to generate single-stranded DNA molecules, captured on Illumina flow cells, amplified *in situ* and finally sequenced for 51 cycles on Illumina HiSeq according to the manufacturer’s instruction.

#### 2.3.3 Raw Data Processing

##### 2.3.3.1 Clean Reads

Raw sequences were generated as clean reads from Illumina NextSeq by real-time base calling and quality filtering.

##### 2.3.3.2 Trimmed Reads

Subsequently, the 3′ adapter sequence was trimmed from the clean reads and the reads with lengths shorter than 8 nt were discarded. As the 5′ adapter was also used as the sequencing primer site, the 5′ adapter sequence is not present in the sequencing reads.

##### 2.3.3.3 Aligned Reads

The trimmed reads (length ≥8 nt) were aligned to the corresponding genome database (Supplementary File SC) using novoalign software (http://www.novocraft.com). sRNA read counts were normalized as tag counts per million (Bullard et al., 2010). Sequences known to be contaminant confounders from RNA isolation procedures were discarded before analysis. Finally, a second dataset was generated for the *E. coli* and OMV samples by excluding the reads that are also present in the LB culture medium sample.

## 3 Results

### 3.1 vsRNAs are Highly Abundant in Bacteria

We analyzed by small RNA-seq the 8−30-nt window of RNA length of the gram-negative *E. coli* MG1655 model organism and 5 other bacterial strains (*P. aeruginosa* PA7, *P. aeruginosa* PAO1, *S. Typhimurium* 14028S, *L. pneumophila* JR32 Philadelphia-1 and *S. aureus* HG001). These bacterial small RNA libraries unveiled the existence of an average of seven million adapter-trimmed reads (min. 5,482,106 reads to max. 9,959,407 reads). The vsRNAs (8–15 nt) accounted for about half of the reads in three strains (*E. coli*, *S. Typhimurium*, *S. aureus*), a quarter in *L. pneumophila* and only 13% in the two *Pseudomonas* (Supplementary Table S2).

**FIGURE 1:**
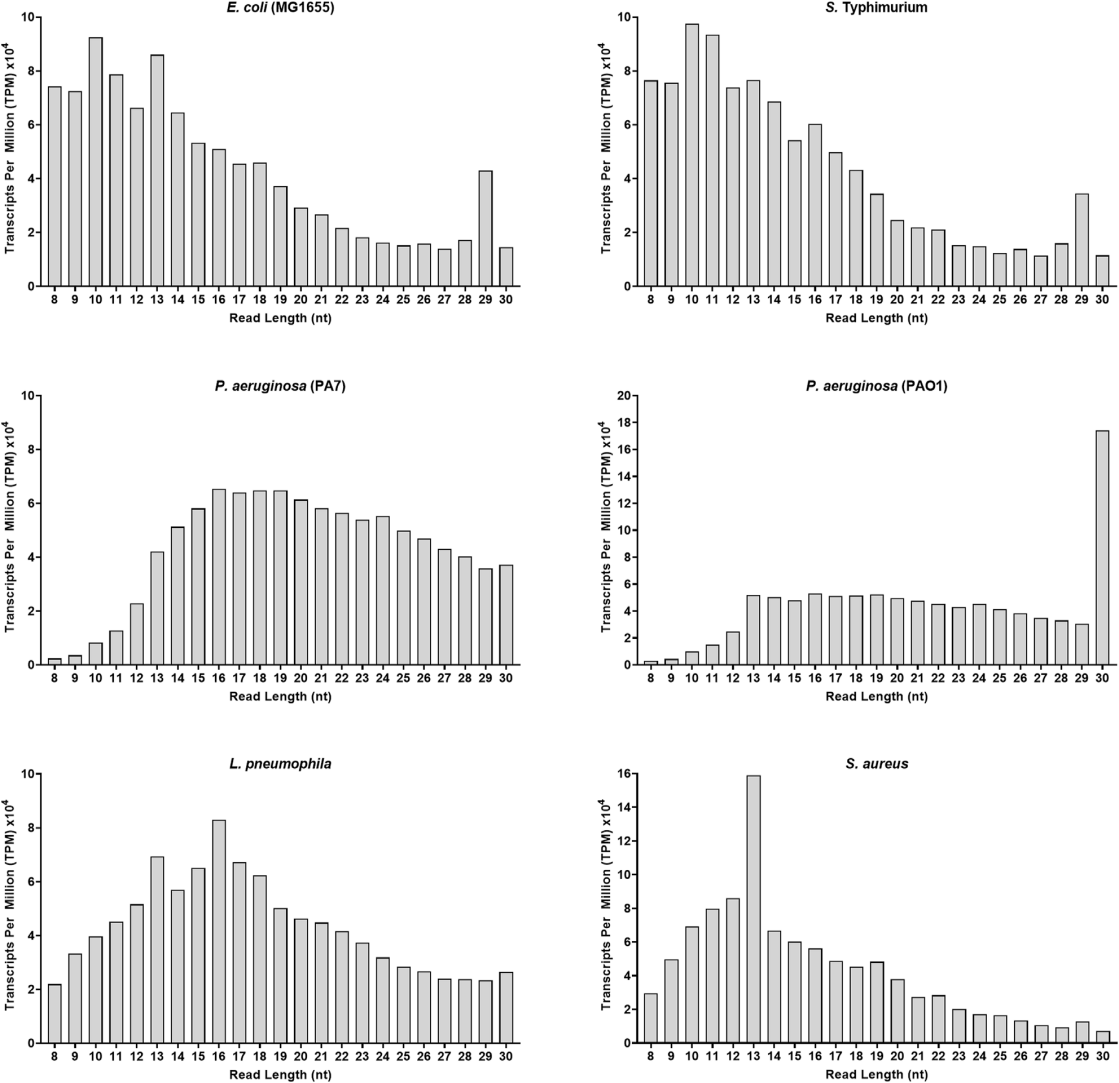
Adapter-trimmed reads length (8–30 nt) distribution in different bacterial strains. Small RNA-Seq analyses in the 8–30 nt window of six bacterial samples. Total adapter-trimmed reads counts were expressed in TPM. The details of the technical approach are described in the section of *Materials and Methods*.

To better highlight the relative abundance of vsRNAs compared to other small RNAs within a sample, we used Transcripts Per Million (TPM) as a second stage of normalization (Zhao et al., 2020). Indeed, except for the two *Pseudomonas*, vsRNAs in all other strains were on average 1.3–3 times more abundant than RNAs above 16 nt (Figure 1). Furthermore, some common patterns in the distribution profiles of sequences were observed between *E. coli* and *S. Typhimurium*, between PA7 and PAO1, and, to a lesser extent, between *L. pneumophila* and *S. aureus* (Figure 1).

### 3.2 vsRNAs Constitute Heterogeneous and Distinct Populations

**FIGURE 2:**
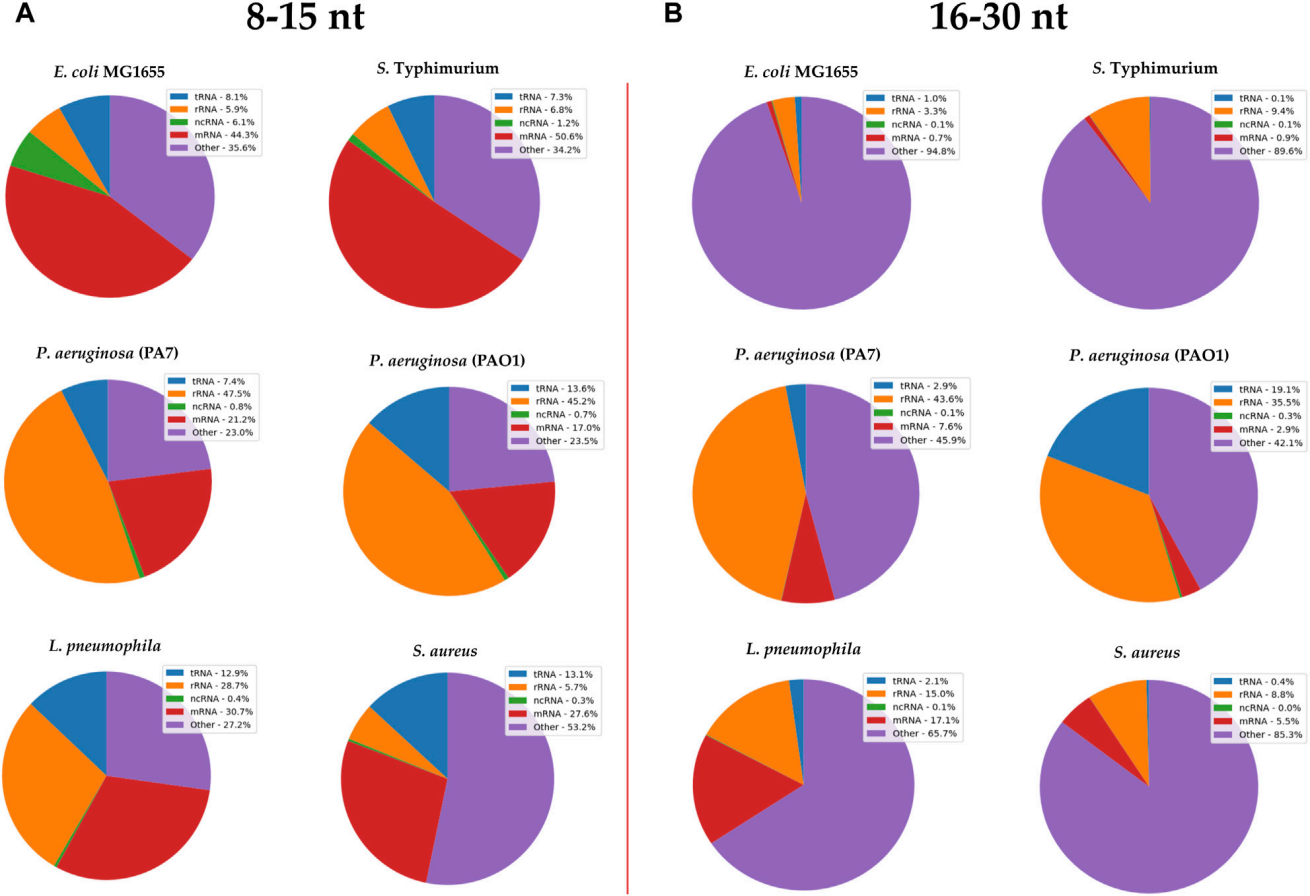
RNA biotypes found in **(A)** 8–15 nt and **(B)** 16–30 nt size libraries from different bacterial strains. Annotated sRNA sequencing reads were classified by RNA-type after alignment to their respective bacterial genomes. The classification of biotypes is made based on the specifications of Ensembl genome database. “ncRNA” refers to all non-coding RNAs except tRNAs and rRNAs; “other” refers to all other RNA fragment that do not derive from a mRNA or ncRNA.

After documenting the existence of vsRNAs in these six bacterial species, we investigated their biotypes, for which we subdivided the data into two separate sets based on RNA length: the first dataset encompassed vsRNAs of 8–15 nt in length [RNAs_(8–15 nt)_] and the other dataset encompassed RNAs of 16–30 nt in length [RNAs_(16–30 nt)_]. Biotype classification was made based on the specifications of Ensembl genome database (Figure 2).

Reads that map to the genome but do not match known RNA biotypes (“other”) represented ∼30% of the RNA_(8–15 nt)_ sequences of the analyzed bacterial strains (Figure 2A). In the RNA_(16–30 nt)_ dataset, this proportion went up to 45% for *Pseudomonas* and even reached 95% in other bacterial strains, including *E. coli* (Figure 2B).

Fragments of mRNA transcripts represented a quarter to half of the bacterial vsRNAs (Figure 2A), but ∼5% of RNA_(16–30 nt)_ sequences (except in *pneumophila*, where they constituted 17% of the total vsRNAs, Figure 2B).

The proportion of tRFs varied between 8% and 13% of vsRNAs, and dropped to less than 3% of the RNA_(16_–_30 nt)_ sequences, except in *Pseudomonas* PAO1 (19%).

The rRNA fragments accounted for nearly half of the biotypes of the two *Pseudomonas* species in both datasets. In *L. pneumophila*, they represented 30% of vsRNAs and only 15% in RNA_16–30 nt_ library. In *E. coli*, *S. typhi* and *S. aureus*, the proportion of rRNAs in both fractions was less than 10%.

In addition, we noticed that there were very few ncRNAs other than tRFs and rRNAs in the two RNA datasets; their highest proportion (only 6%) was found in *E. coli*, while in other species they rarely exceeded 1% (Figure 2).

Although the sRNA profile of *E. coli*, *S. Typhimurium* and that of the two *Pseudomonas* is similar, RNA profiling and clustering data suggest that vsRNA biotypes are distinct and varied within and across bacterial species.

### 3.3 vsRNAs are Also Found in *E. coli*-Derived Outer Membrane Vesicles

**FIGURE 3:**
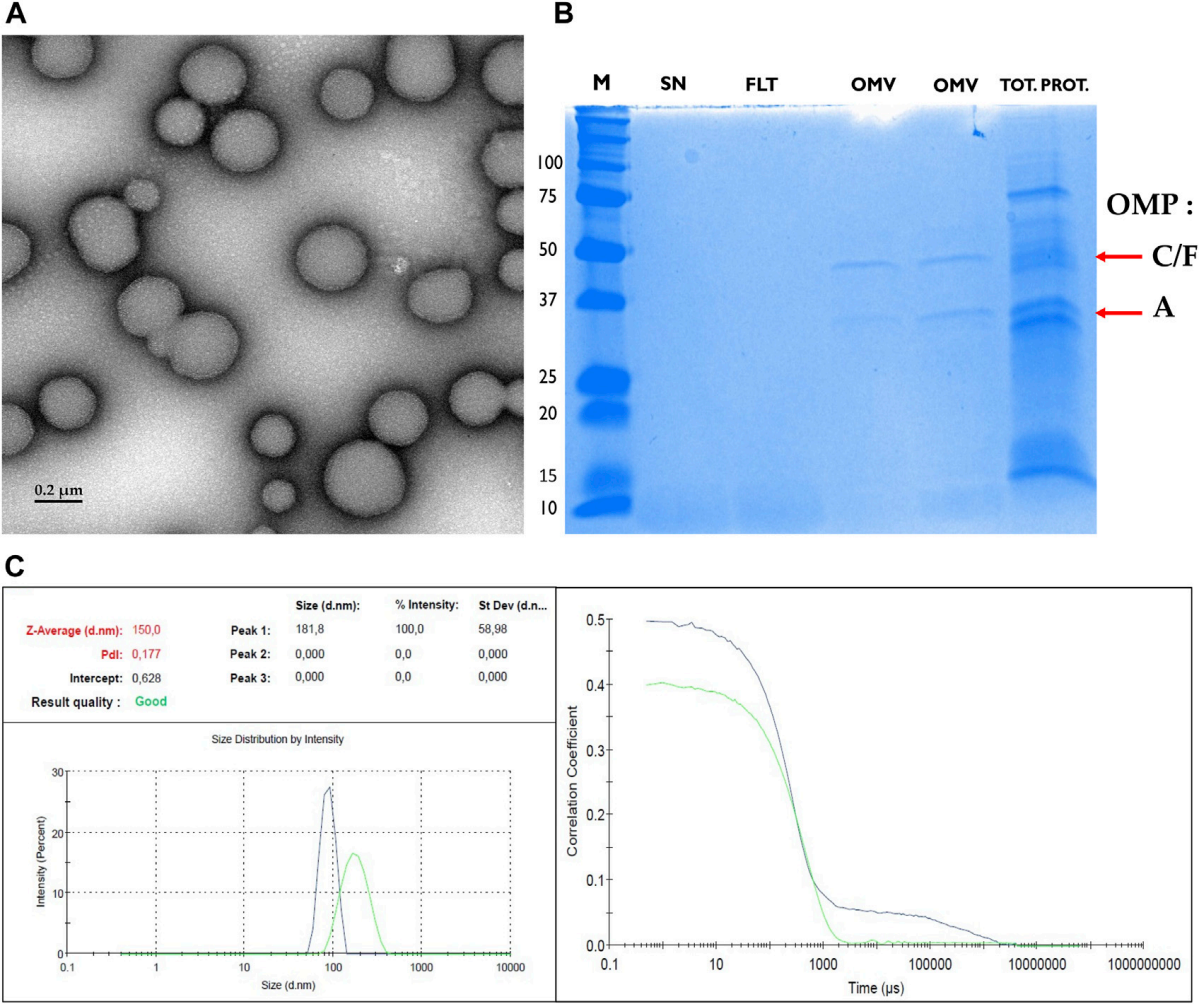
Biophysical characterization of OMVs. **(A)** Transmission electron microscopy image of purified *E. coli* outer membrane vesicle (OMV) showing size and morphology. Scale bar, 200 nm. **(B)** Coomassie blue-stained SDS-PAGE of OMVs from cultures of *E. coli* K12 MG1655 shows successful purification of OMVs depleted from cellular debris. From left to right we illustrate the molecular weight ladder (M, in kDa), the supernatant (SN), the filtrate (FLT), two samples of OMV: the first being isolated using ultracentrifugation approach and the second using the Exobacteria kit, and at the very right the total protein fraction (TOT. PROT.). The successful enrichment of OMVs with the ultracentrifugation or the Exobacteria kit was confirmed by the presence of two bands which were verified to correspond to the major Outer Membrane Proteins (OMPs) and OMV marker proteins OmpF/C (40 kDa) and OmpA (35 kDa) (Horstman and Kuehn, 2000; Daleke-Schermerhorn et al., 2014). The positions of OMPs F, C, and A are indicated by red arrows. **(C)** Dynamic Light Scattering analysis of the hydrodynamic size of *E. coli* OMVs. The Intercept value: 0.628 = the signal-to-noise ratio = ideal signal between = (0.6–1); the Polydispersity Index (PdI): 0.177 = diversity of size distribution = indicate if samples are suitable or not: (0.05–07); The Z-average :150 = hydrodynamic parameter insensitive to noise = average size of a particle size distribution.

Since OMVs are released by Gram-negative and Gram-positive bacteria, we next focused on the reference bacterial strain *E. coli* K-12 MG1655, isolated its derived OMVs and analyzed their respective content in vsRNAs. Transmission electron microscopy confirmed the presence of OMVs displaying spherical shape with an average diameter of ∼180 nm (Figure 3A). Isolated bacterial OMVs subjected to SDS-PAGE analysis and Coomassie blue staining revealed the presence of major vesicle protein bands corresponding to the known outer membrane proteins (OMP) A, C, and F (Figure 3B), which are common OMV markers. The purity of the isolated OMVs was consequently confirmed by the absence of bacterial debris. We have additionally analyzed the biophysical features of the OMVs by dynamic light scattering (DLS, Figure 3C). The DLS data corroborated the microscopy data and showed an average particle size of OMVs around 150 nm. The polydispersity index (PdI) values were 0.177 indicating size heterogeneity in the OMVs population (monodisperse samples have generally a PDI <0.05).

**FIGURE 4:**
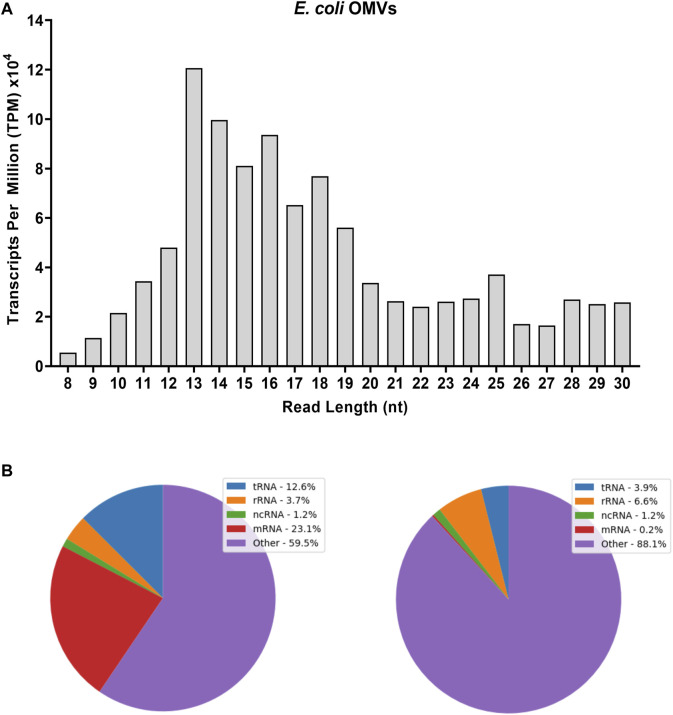
Adapter-trimmed reads length (8–30 nt) and biotypes distribution in *E. coli*-derived OMVs. Small RNA-Seq analyses in the 8–30 nt window of OMV sample. **(A)** Total adapter-trimmed read counts expressed in TPM. **(B)** Annotated sRNA sequencing reads were classified by RNA-type after alignment to *E. coli* genome. “ncRNA” refers to all non-coding RNAs except tRNAs and rRNAs; “other” refers to all other RNA fragment that do not derive from a mRNA or ncRNA. Purified OMVs were obtained with the Exobacteria kit (For details, see *Materials and Methods*).

RNA-seq analysis of *E. coli*-derived OMVs was performed along with that of the other bacterial strains described above. Regarding the variety of biotypes and their abundance, the OMVs were as rich in RNA as the bacterial samples (Figure 4A). The vsRNAs from OMVs accounted for one third of the 7.2 million reads identified in the 8–30 nt window (Supplementary Table S2). The adapter-trimmed reads length distribution in OMVs was different from those in *E. coli* from which they derive (Figure 4A vs. Figure 1), although the profile of their biotypes was quite similar in distribution (Figure 4B). OMV RNA sequences that did not correspond to any known biotype represented 60% and 90% of the reads in the RNAs_(8–15 nt)_ and RNAs_(16–30 nt)_ datasets, respectively (Figure 4B).

### 3.4 Selective Loading of vsRNAs, Especially tRFs, in Outer Membrane Vesicles

**FIGURE 5:**
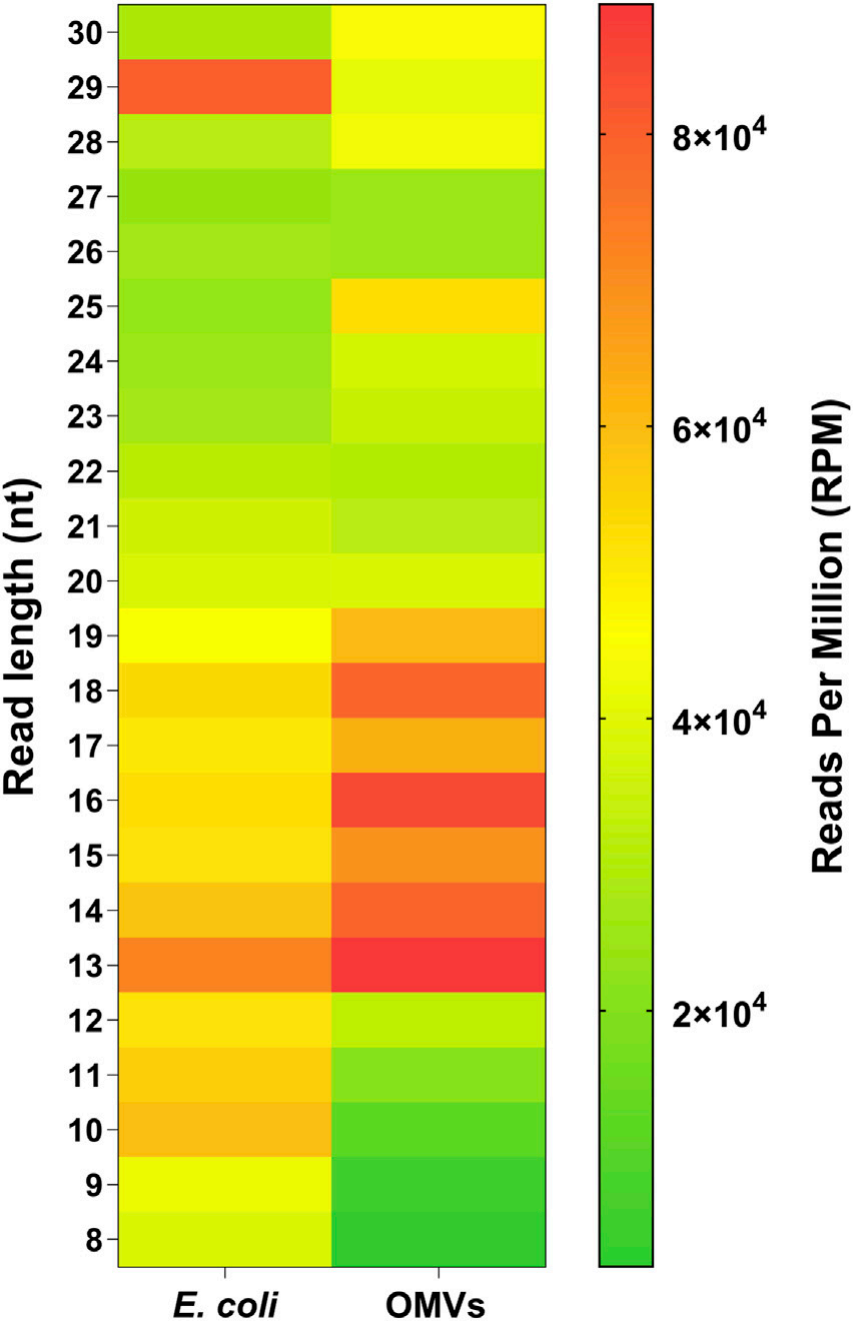
Selective loading of RNA in OMVs. Heatmap showing RNA distribution between *E. coli* and OMVs in relation to sequence length (8–30 nt). The total reads counts were normalized in reads per millions (RPM). Purified OMVs were obtained with the Exobacteria kit (For details, see *Materials and Methods*).

To help elucidate the dynamics of RNA distribution between *E. coli* and their OMVs, we normalized the RNA-seq data to reads per million (RPM). Intracellular RNAs and those of OMVs were derived from the same culture and were processed in an identical manner. Heatmap representation of these data illustrate a selective loading of vsRNAs in OMVs with a particular enrichment of RNAs between 13 and 19 nt and, to a lesser extent, RNAs of 23–25 nt in length (Figure 5). RNA fragments of 8–12 nt and of 29 nt were 2- to 15-fold more abundant in bacteria than in their OMVs. On the other hand, the distribution pattern seems to be similar for the 20–22 and 26–27 nt RNAs.

Examination of the 20 most abundant sequences in *E. coli* and their OMVs (Tables 1, 2) revealed tRFs as a major biotype of interest, since the other sequences were composed mainly of short mRNA fragment reads. Ile-tRF and Ala-tRF were among the most abundant tRFs found in both *E. coli* and OMVs; Ile-tRF was 7 and 4 times more abundant than Ala-tRF, respectively. However, Ile-tRF and Ala-tRF were 9 and 18-fold enriched in OMVs compared to bacteria, suggesting a preferential loading of these small tRFs in OMVs (Tables 1, 2).

**TABLE 1:** Top20 of the most abundant reads in *E. coli* sample.

Rank	Reads	Sequences	Possible origins
1	x9295	TCCGATGTAGT	mRNA fragment
2	x8150	AGGCTTGTAGCTC	tRNA-Ile
3	x7776	TCCGATGTAGTGTA	mRNA fragment
4	x7729	GCGGATTTAGTTA	mRNA fragment
5	x6509	AGGCTTGTAGCTT	tRNA-Ile
6	x5062	AGGCTTGTAGCTA	tRNA-Ile
7	x3918	ATTCGGGTCTTGTA	mRNA fragment
8	x3632	TTCGGGTCTTGTA	mRNA fragment
9	x3429	TAAGGAGTGTGTA	mRNA fragment
10	x3428	CTTGTGGCGTA	mRNA fragment
11	x3372	TCCGATGTAGC	mRNA fragment
12	x3017	TCCGATGTAG	mRNA fragment
13	x2810	TCCGATGTAGTGT	mRNA fragment
14	x2733	CATTCGGGTCTTGTA	mRNA fragment
15	x2693	AATTCCTAGTA	mRNA fragment
16	x2611	GGGGCTATAGCTC	tRNA-Ala
17	x2605	TTGGCGGA	No hit
18	x2572	TCCGATGTAA	mRNA fragment
19	x2560	TCCGATGTAGA	mRNA fragment
20	x2498	CTAAGGAGTGTGTA	No hit

**TABLE 2:** Top20 of the most abundant reads in OMVs sample.

Rank	Reads	Sequences	Possible origins
1	78450	AGGCTTGTAGCTC	tRNA-Ile
2	73912	AGGCTTGTAGCTT	tRNA-Ile
3	23705	AGGCTTGTAGCTA	tRNA-Ile
4	22218	GGGGCTATAGCTC	tRNA-Ala
5	22074	GGGTCTGTAGCTT	23S rRNA
6	21957	GGGTCTGTAGCTC	23S rRNA pseudouridylate synthase B
7	18375	GGGGCTATAGCTT	tRNA-Ala
8	12052	CCGGGAGGAGCTCT	mRNA fragment
9	8594	ACTAGGGATCGGGTG	mRNA fragment
10	7661	CTCTTGTAGACCGTT	mRNA fragment
11	7024	GGGTCTGTAGCTA	23S rRNA pseudouridylate synthase B
12	6184	CGGCACGTAGCGT	tRNA-Pro
13	6044	GGGGCTATAGCTA	tRNA-Ala
14	5508	CTAGGGATCGGGTG	mRNA fragment
15	5280	CGAGACCTTAACCT	mRNA fragment
16	4318	ACCAGGAGTGGAGCT	mRNA fragment
17	4301	AGGCTTGTAGCT	tRNA-Ile
18	4278	CGGCACGTAGCGC	tRNA-Pro
19	4023	CCGGGAGGAGCTCA	mRNA fragment
20	3202	GCTGGCTCCGG	rRNA small subunit methyltranserase D

Furthermore, previous analysis of bacterial strain DH5α, which belongs to the same clade as the *E. coli* MG1655 reference strain, showed that tRFs represented more than half of the top 20 most abundant tRFs, with Ile-tRF and Ala-tRF in pole position (Supplementary Table S3). In the other five bacterial strains investigated (except for *S. Typhimurium*), the most abundant sequences also included tRFs (data not shown, GEO accession number: GSE200758; Bioproject accession number: PRJNA826503).

### 3.5 tRFs Probably Result From Specific Processing

tRF sequences align to the 5′ side of mature tRNAs and are thus classified as tRF-5 (Supplementary File SD). The most abundant tRFs identified were predominantly 13 nt in length. tRNA processing into tRFs seems to involve cleavage after the sequence motif “TAGC” located on the D-arm, at the beginning of the D-loop, where the 13th nt usually carried a pseudouridine (*Ψ*) highly conserved among species (Chan and Huang, 2009; Chen et al., 2010).

The mRNA and rRNA fragments randomly mapped to different positions on their original genes in contrast to the tRFs fragments, which were more consistently localized to unique positions. These observations suggest that bacterial tRFs may originate from specific processing events rather than random degradation.

### 3.6 tRFs are Thermodynamically Stable

We then analyzed tRFs secondary structure, as well as that of other RNA biotypes, using RNAfold 2.4.18 (Mathews et al., 2004). With the sequences being redundant or differing by some nucleotides at their 3′ end, we have chosen the most representative ones in the Top20 of the most abundant sequences.

**FIGURE 6:**
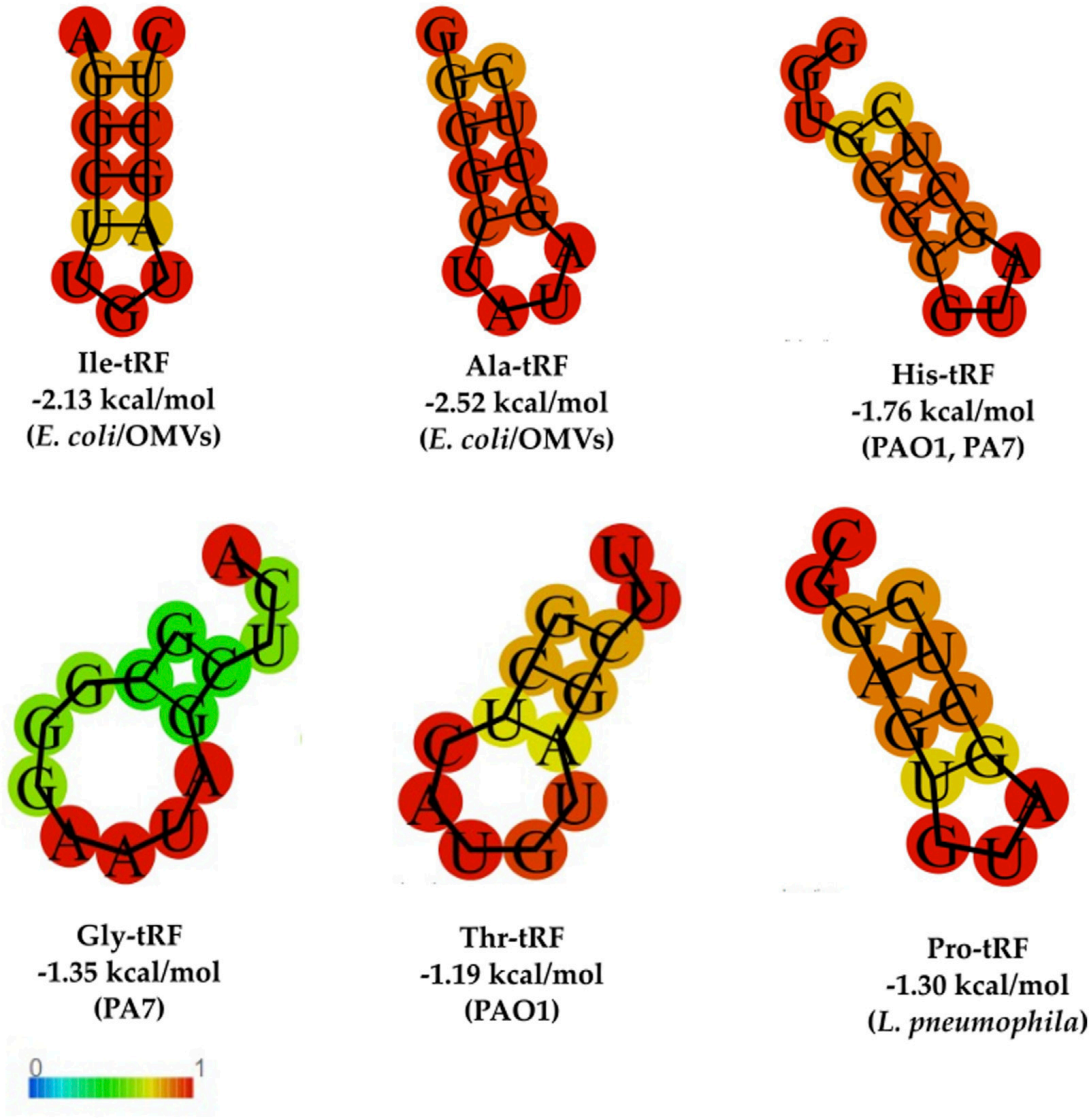
Illustration of the secondary structure of the six most abundant tRFs. RNAfold was used for RNA secondary structure prediction. The values in kcal/mol represent the minimum free energies. The probability of the base pairs is color-coded 0 to 1, with the red color corresponding to higher confidence. All oligonucleotides analyzed have at least 2 G-C base pairs that provide for stem loop stability.

Figure 6 shows that tRFs are thermodynamically stable with stem-loop structure (hairpin). Their free energy of thermodynamic ensemble varied between −2.52 and −1.19 kcal/mol. These stable stem-loop structures were also found in *E. coli*, their OMVs and in other bacterial strains (e.g., *L. pneumophila* PAO1 and PA7). *S. aureus* Pro-tRF exhibited an unstable circular structure (−0.40 kcal/mol) in contrast to its *L. pneumophila* analog (hairpin, −1.30 kcal/mol) underlining sequence-specific structure variations.

Sequences from other biotypes (rRNA, mRNA) showed mostly unstructured (circular) molecular organization with low minimum free energy (Supplementary Figure S1) except for three sequences that have adopted stable stem-loop conformations: two fragments derived from the 16S rRNA (in PAO1, PA7, and *L. pneumophila*) and one fragment derived from a mRNA (in OMVs).

Taken together, these findings suggest that bacterial tRFs are produced upon specific processing of tRNAs and have superior thermodynamic stability compared to the other vsRNAs.

### 3.7 tRFs are Predicted to Target Several mRNAs With Diverse Functions

The enrichment of vsRNAs, particularly tRFs, in OMVs suggests a potential role in intra- and inter-species communications and prompted us to speculate that bacterial tRFs might function as their eukaryotic counterparts or analogous miRNAs. Potential human target mRNAs of these tRFs were identified with DIANA-microT (Paraskevopoulou et al., 2013) and BLASTN (Altschul et al., 1990), and consolidated with RNAhybrid (Krüger and Rehmsmeier, 2006). The results are summarized in Supplementary File SE. The enrichment analysis was performed using PANTHER Classification System (Mi et al., 2013) for the significant human targets. All terms related to the three aspects of GO (Molecular Function, Biological Process, Cellular Component) are grouped in Supplementary File F.

**FIGURE 7:**
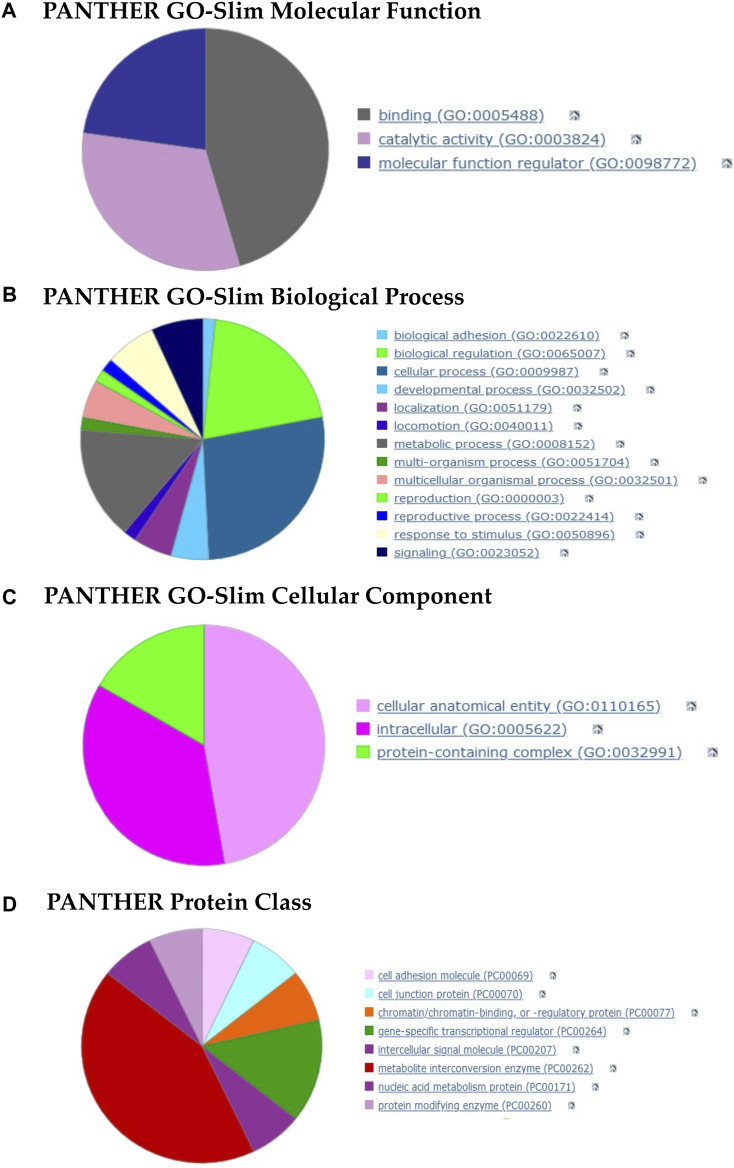
Pie charts of gene function analysis based on the tRFs targets and performed with PANTHER. **(A–C)** Analysis of the 3 GO aspects (molecular function, biological process, cellular component). **(D)** PANTHER Index terms describing protein classes (panther classification). The detailed terms for each category are summarized in the Supplementary File SC.

The molecular-level activities performed by tRF-targeted gene products were related, among other terms, to metal ion binding (GO: 0046872, *p*-value: 8.91E-04), BH3 domain binding which is a potent death domain present in Bcl-2 family members (GO: 0051434; *p*-value: 8.79E-03), piRNA binding (GO: 0034584, *p*-value: 6.29E-03) and MAP kinase kinase kinase activity (GO: 0004709; *p*-value: 3.47E-02). Figure 7A summarizes the three molecular functions to which all the activities belong.

The overrepresented GO terms for Biological Process aspect (more than half) were related to biological regulation, cellular and metabolic processes (Figure 7B). More specifically, we found terms such as cell differentiation (GO: 0030154, *p*-value: 6.21E-03), regulation of cellular response to stress (GO: 0080135, *p*-value: 8.02E-03), B cell chemotaxis (GO: 0035754, *p*-value: 8.79E-03) and regulation of macrophage colony-stimulating factor production (GO: 1901256; *p*-value: 8.79E-03).

The cellular component in which these targets perform their function were cellular anatomical entities and protein-containing complexes (Figure 7C). We found, among other terms, the Bcl-2 family protein complex (GO: 0097136, *p*-value: 8.79E-03), cell body membrane (GO: 0044298, *p*-value: 3.96E-02) and RNA-directed RNA polymerase complex (GO: 0031379; *p*-value: 5.03E-03).

Lastly, the protein class terms (Figure 7D) associated with these targets were mainly intercellular signal molecules, cell adhesion molecules, metabolite interconversion enzymes and gene-specific transcriptional regulators.

Overall, the enrichment analyses suggest that tRFs may not target a specific pathway and seem to be capable of assuming various and multiple functions in the hosts.

## 4 Discussion

The post-genomic era of sRNA discovery is marked by a paradigm shift in our understanding of transcriptional and post-transcriptional gene regulation in bacteria. In a little more than a decade, we went from about fifty well-characterized sRNAs (Hershberg et al., 2003), mainly in *E. coli* and *Salmonella*, to thousands (Wang et al., 2016), and thousands more to come (Barquist and Vogel, 2015). The discovery of vsRNAs, including tRFs, broadens the scope and diversity of bacterial sRNAs, and prompts us to reconsider the biological relevance of sRNAs shorter than 16 nt and that of the dogmatic length threshold above which sRNAs are to be considered as biologically important, as we previously discussed (Plante et al., 2012; Lambert et al., 2019; Diallo and Provost, 2020; Lambert et al., 2021).

The length distribution of bacterial sRNAs is usually comprised between 50 and 250 nt (Hershberg et al., 2003). The discovery and characterization of small functional RNAs, such as miRNAs (19–22 nt) and tRFs (14–40 nt) in eukaryotes (Lee et al., 2009; Bartel, 2018), did not lead to the systematic search for potential sequence analogies in bacteria. For instance bacterial miRNAs have received little attention, although their existence has been suggested (Lee and Hong, 2012; Kang et al., 2013; Dang et al., 2019). Bacterial tRFs have recently gained interest due to their discovery in extracellular vesicles and their potential roles in host immune modulation (Li and Stanton, 2021).

The pursuit of this paradigm shift may pose new technical challenges, even though the use of aligners, such as Novoalign, showed higher sensitivity towards short reads (Li and Homer, 2010; Thankaswamy-Kosalai et al., 2017). Their assembly as well as their annotation remains an algorithmic challenge (Xie et al., 2014). Depending on the research goals and purpose of the RNA-seq analyses, the high number of vsRNAs may affect the depth and sensitivity of the sequencing, influence data accuracy and impede the discovery of low abundance RNAs. Preservation in the samples of rRNAs, which are the main cellular RNAs by mass, may have a similar effect. Nonetheless, total RNA library may allow the detection of additional RNAs and has the advantage of capturing some RNA subgroups which would pass under the radar when applying a rRNA depletion (Guo et al., 2015; Zhao et al., 2018).

Our RNA-seq study supports the widespread expression of vsRNAs in gram-positive and gram-negative bacteria, expanding on their existence in higher organisms (Lambert et al., 2021). Each of our sRNA datasets [RNAs_(8−15nt)_ and RNAs_(16−30nt)_] displayed heterogeneous and distinct content of RNAs. We noted an over-representation of mischaracterized RNA, which was neither classical ncRNAs nor mRNAs in the 16–30-nt size window in *E. coli, S. Typhimurium, L. pneumophila* and *S. aureus*. These unknown non-coding RNAs may correspond to unannotated RNA fragments, 5’/3′UTR or might be part of new genetic elements (Stav et al., 2019). In both eukaryotes and prokaryotes, the biotypes that account for the highest proportion of RNA reads are usually rRNAs and tRNAs but also their derivatives (fragments), mainly because of their abundance in cells (Kennell, 1968; Baracchini and Bremer, 1987; Palazzo and Lee, 2015). The results, however, showed a different profile without predominance of these abundant RNAs (Figure 2) unless we zoom on the most abundant sequences (Tables 1, 2). Regarding tRNA fragments, the upper limit of the libraries (30 nt) eliminates by default the very abundant tRNA halves (tiRs) which are generally 31–40 bases long (Saikia et al., 2012), thus limiting the overall proportion of tRFs. Although our study is the first to examine libraries containing bacterial sRNAs as short as between 8 and 15 nt in length, meaningful comparisons with other studies, in terms of biotypes, are hindered due to differences in sequencing strategies and analytical tools used (Ghosal et al., 2015; Blenkiron et al., 2016).

Initially proposed to mediate cellular waste disposal (Johnstone et al., 1987), extracellular vesicles are now recognized as a key player in intercellular communication, with their RNA content being at the center stage (Ainsztein et al., 2015; Hessvik and Llorente, 2018). In bacteria, extracellular vesicle (EVs) such as OMVs are gradually unraveling their secrets and their RNA content is being scrutinized more than ever (Ghosal et al., 2015; Ho et al., 2015; Sjöström et al., 2015; Blenkiron et al., 2016; Koeppen et al., 2016). Our study expands the repertoire of sRNAs present in bacteria and bacterial OMVs to species as short as 8 nt in length, some of which do not correspond to known biotypes; the question of their redundancy and potential role remains to be elucidated. It may not be prudent to dismiss these mischaracterized ncRNAs before their detailed characterization, as very little goes to waste in cells (De Lay and Garsin, 2016). Moreover, their unique abundances, characteristics and properties could be useful as biomarkers for the prediction of infections, their stages and prognosis (Quinn et al., 2015).

It should be noted that the use of complex media, such as LB, considerably contaminates the sequencing data. In our study, around 41% of the reads from *E. coli* or OMV RNA fractions mapped to LB medium components (Supplementary Table S4), making their sequencing and removal from datasets mandatory when studying sRNAs (Pavankumar et al., 2012; Ghosal et al., 2015). This should be applied to any microbial media containing yeast extracts, bacto-peptone or other components from living organisms. In the 8–30-nt window of RNA length under study, the percentages of cleaned reads mapping to the *E. coli* genome in bacteria and their OMVs (after removal of the reads identical to LB medium components), were 34.1% and 23%, respectively. Working with the same strain, but focusing on a different window of RNA length (15–50 nt), Ghosal et al. (2015) reported mapping of 97.7% and 34.7%, respectively. Shifting the lower sequencing window limit to 8 nt may increases the probability of contaminating the data with reads from the culture medium if their removal is not included in the pipeline. However, the inclusion of smaller sequences does not alter the quality of the data collected.

Our study corroborates and expands on previous findings suggesting the presence of tRFs in bacterial OMVs (Li and Stanton, 2021). Similar to eukaryotic extracellular vesicles and exosomes, there seems to be a specific mechanism for sorting and transferring gene regulatory sRNAs from bacteria to human host cells through OMVs, the details of which remain to be elucidated (Diehl et al., 2012; Laffont et al., 2013; Laffont et al., 2016; Hessvik and Llorente, 2018). Here, we observed that the length of the sRNAs and by extrapolation their belonging biotype may be a key factor in packaging (Figure 5). Moreover, examination of the particular enrichment of tRFs in OMVs highlighted the presence of conserved short motifs (“TAGCT”) that may participate (in association with proteins) in the process of sorting into OMVs. A similar process has been characterized with the loading of miRNAs into exosomes (Villarroya-Beltri et al., 2013). Eukaryotic tRFs are catalogued in several databases (Kumar et al., 2015; Zheng et al., 2016; Pliatsika et al., 2018). Although some tools for predicting their mRNA targets are available (Li et al., 2021; Xiao et al., 2021; Zhou et al., 2021), none of them support bacterial tRFs and most of them are set for sRNAs longer than 16 nt. Of note, miRNA prediction tools (Singh, 2017) can be employed for the prediction of tRF mRNA targets, only if we assume functional and mechanistic analogies (Kumar et al., 2014). This represents a serious limitation to the study and characterization of tRFs and, more globally, of vsRNAs (shorter than 16 nt in length).

To circumvent these bioinformatics limitations to target identification—the sequence length of vsRNAs may increase their probability of binding RNA targets—we have used heuristic search method combining different tools (Altschul et al., 1990; Krüger and Rehmsmeier, 2006; Mi et al., 2013; Paraskevopoulou et al., 2013). The use of PANTHER, for instance, has enabled the identification of multiple potential targets for vsRNAs, without specific pathway domination. Additional studies are required to confirm these vsRNA-target interactions, which may be involved in modulating host cell gene expression and function, and host-pathogen relationship.

## Data Availability

The datasets presented in this study can be found in online repositories. The names of the repository/repositories and accession numbers GEO accession: GSE200758, Bioproject accession: PRJNA826503 can be found in the article/Supplementary Material.
