# Supplementary material for: RNA Sequencing Unveils Very Small RNAs With Potential Regulatory Functions in Bacteria
Source: Front Mol Biosci. 2022 Jun 3;9:914991. doi: 10.3389/fmolb.2022.914991 (PMC9203972; doi:10.3389/fmolb.2022.914991)
Supplement: Supplementary file 1 [file DataSheet1.ZIP › Supplementary_Files/Supplementary_File_A_Workflow/Supplementary_File_A_workflow.docx]

**Experiment Workflow**

**Flowchart of Data Analysis**
