# Supplementary material for: RNA Sequencing Unveils Very Small RNAs With Potential Regulatory Functions in Bacteria
Source: Front Mol Biosci. 2022 Jun 3;9:914991. doi: 10.3389/fmolb.2022.914991 (PMC9203972; doi:10.3389/fmolb.2022.914991)
Supplement: Supplementary file 1 [file DataSheet1.ZIP › Supplementary_Files/Supplementary_File_C_GenomeDatabase/Supplementary_File_C_Genome Database.docx]

**Idrissa Diallo et al.,**

**File C.**

| **Species** | **GenBank assembly accession** |
| --- | --- |
| Legionella pneumophila | **GCA_002934185** |
| Pseudomonas aeruginosa (PA7) | **GCA_000017205** |
| Pseudomonas aeruginosa (PAO1) | **GCA_000006765** |
| Staphylococcus aureus | ***GCA_001018655*** |
| Saccharomyces cerevisiae | **GCA_000146045.2** |
| Salmonella enterica | **GCA_001558355** |
| Escherichia coli | **GCA_000005845** |
| - All tRNA obtained from GtRNAdb (<http://gtrnadb.ucsc.edu/>) - All ncRNA/mRNA obtained from Ensembl Bacteria using above accession numbers | |
