# Supplementary material for: RNA Sequencing Unveils Very Small RNAs With Potential Regulatory Functions in Bacteria
Source: Front Mol Biosci. 2022 Jun 3;9:914991. doi: 10.3389/fmolb.2022.914991 (PMC9203972; doi:10.3389/fmolb.2022.914991)
Supplement: Supplementary file 1 [file DataSheet1.ZIP › Supplementary_Files/Supplementary_File_B_Sample_QC/Supplementary_File_B_Sample_QC.pdf]

Sample Quality Control

1.

RNA-QC Report

1. RNA Quantity and Purity Test by NanoDrop ND-1000

Sample ID	OD260/280 Ratio	OD260/230 Ratio	Conc. (ng/µl)	Volume (µl)	Quantity (ng)
1 : E. coli K12 MG1655 – Reference (Exponential phase)	1.8	1.98	124.12	15	1861.8
~~2 : E. coli K12 MG1655 – Stationary phase~~	~~1.84~~	~~2.05~~	~~144.67~~	~~15~~	~~2170.05~~
~~3 : E.coli K12 MG1655 + Chloramphenicol (Inhibition of Protein synthesis)~~	~~1.79~~	~~1.97~~	~~100.55~~	~~15~~	~~1508.25~~
~~4 : E. coli K12 MG1655 +Rifampicin (Inhibition of RNA synthesis)~~	~~1.82~~	~~2.03~~	~~104.89~~	~~15~~	~~1573.35~~
~~5 : E. coli K12 MG 1655 - EM1277 ; rne-3071 ts (inhibition of RNase E)~~	~~1.81~~	~~2.04~~	~~118.15~~	~~15~~	~~1772.25~~
~~6 : E. coli K12 MG 1655 – △ssrA (Deletion of tmRNA)~~	~~1.84~~	~~2.07~~	~~136.68~~	~~15~~	~~2050.2~~
7 : P. aeruginosa PAO1	1.86	2.11	136.85	15	2052.75
8 : P.aeruginosa PA7	1.83	2.01	113.35	15	1700.25
9 : S. aureus	1.89	2.03	113.67	15	1705.05
10 : L. pneumophila	1.84	1.82	93.71	15	1405.65
11 : S. thyphimurium	1.83	1.94	112.08	15	1681.2
12 : RNA OMV WT E. coli Reference	1.62	1.52	36.13	15	541.95
~~13 : RNA E. coli OMV △ssrA~~	~~1.53~~	~~1.47~~	~~23.13~~	~~15~~	~~346.95~~
14 : LB Culture medium	1.82	1.96	149.13	15	2236.95

2. RNA Integrity and gDNA contamination test by Denaturing Agarose Gel Electrophoresis

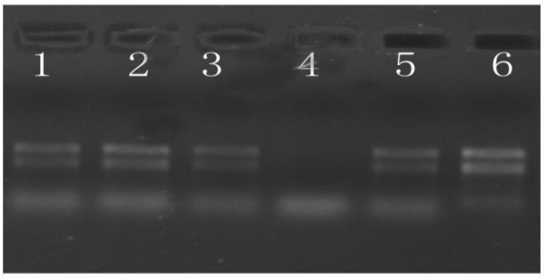

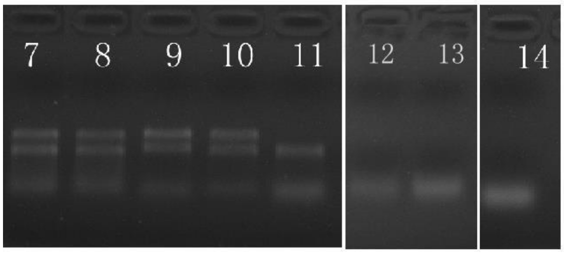

2. Quality Assessment of Sequencing Library

Sequencing library was determined by Agilent 2100 Bioanalyzer using the Agilent DNA 1000 chip kit (Agilent, part # 5067-1504)

Sample Name	Size (bp)	Conc. (ng/µl)	Conc. (nmol/L)	Volume (µl)**	Total Amount (ng)
1 : E. coli K12 MG1655 – Reference (Exponential phase)	147	1.82	18.8	10	18.2
~~2 : E. coli K12 MG1655 – Stationary phase~~	~~148~~	~~1.04~~	~~10.6~~	~~10~~	~~10.4~~
~~3 : E.coli K12 MG1655 + Chloramphenicol (Inhibition of Protein synthesis)~~	~~146~~	~~2.85~~	~~29.6~~	~~10~~	~~28.5~~
~~4 : E. coli K12 MG1655 +Rifampicin (Inhibition of RNA synthesis)~~	~~146~~	~~1.16~~	~~12.1~~	~~10~~	~~11.6~~
~~5 : E. coli K12 MG 1655 - EM1277 ; rne-3071 ts (inhibition of RNase E)~~	~~146~~	~~2.06~~	~~21.4~~	~~10~~	~~20.6~~
~~6 : E. coli K12 MG 1655 – △ssrA (Deletion of tmRNA)~~	~~145~~	~~1.06~~	~~11.1~~	~~10~~	~~10.6~~
7 : P. aeruginosa PAO1	146	1.59	16.4	10	15.9
8 : P.aeruginosa PA7	146	1.57	16.3	10	15.7
9 : S. aureus	145	1.40	14.6	10	14.0
10 : L. pneumophila	147	2.16	22.3	10	21.6
11 : S. thyphimurium	147	1.34	13.8	10	13.4
12 : RNA OMV WT E. coli Reference	147	1.40	14.4	10	14.0
~~13 : RNA E. coli OMV △ssrA~~	~~147~~	~~1.17~~	~~12.1~~	~~10~~	~~11.7~~
14 : LB Culture medium	147	1.09	11.2	10	10.9

**The libraries were adjusted to 10nM before cluster generation.

Quality control analysis on an Agilent 2100 Bioanalyzer

Sample 1. E. coli K12 MG1655:

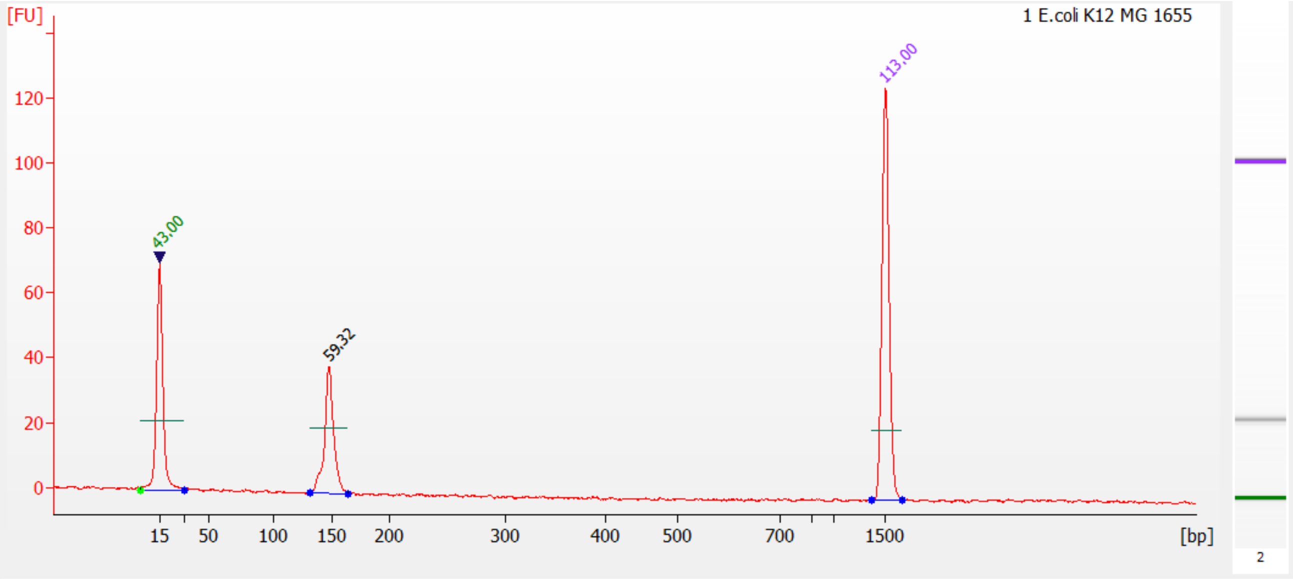

		Size [bp]	Conc. [ng/µl]	Molarity [nmol/l]	Observations
▶	◀	15	4.20	424.2	Lower Marker
2		147	1.82	18.8	
3	▶	1,500	2.10	2.1	Upper Marker

Sample 2 E. coli K12 MG1655:

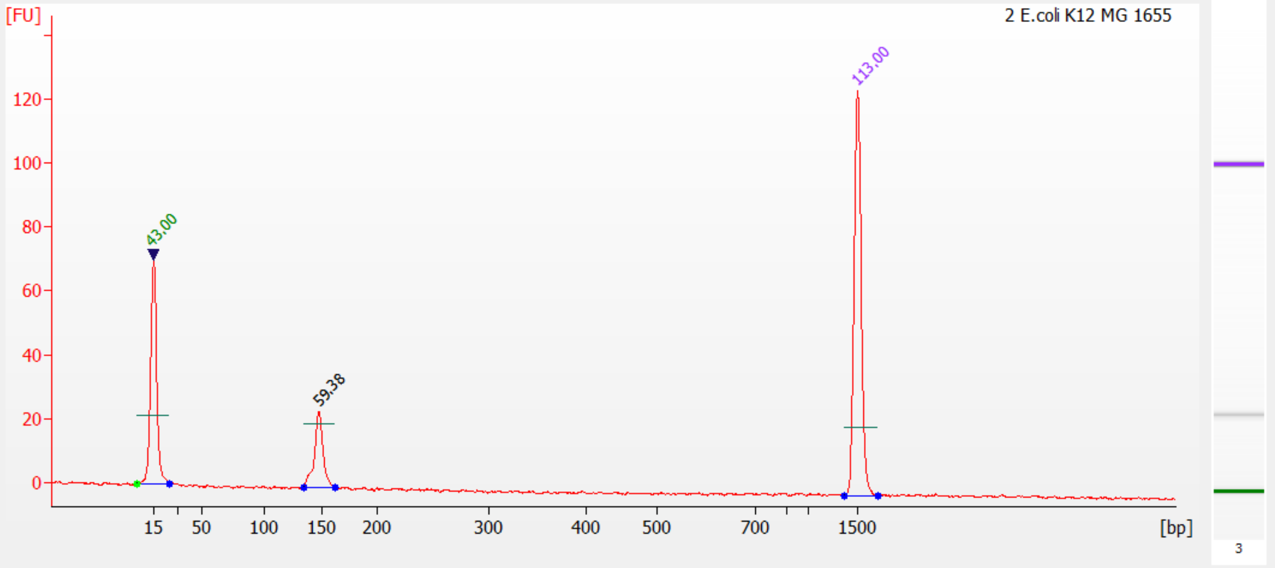

		Size [bp]	Conc. [ng/µl]	Molarity [nmol/l]	Observations
▶	◀	15	4.20	424.2	Lower Marker
2		148	1.04	10.6	
3	▶	1,500	2.10	2.1	Upper Marker

Arraystar, Inc. 9430 Key West Avenue Suite 128
Rockville, Maryland 20850. USA
Tel: 240-314-0388
Fax: 240-238-9860
Email: support@arraystar.com
Website: www.arraystar.com

Sample ~~3. E. coli K12 MG1655:~~

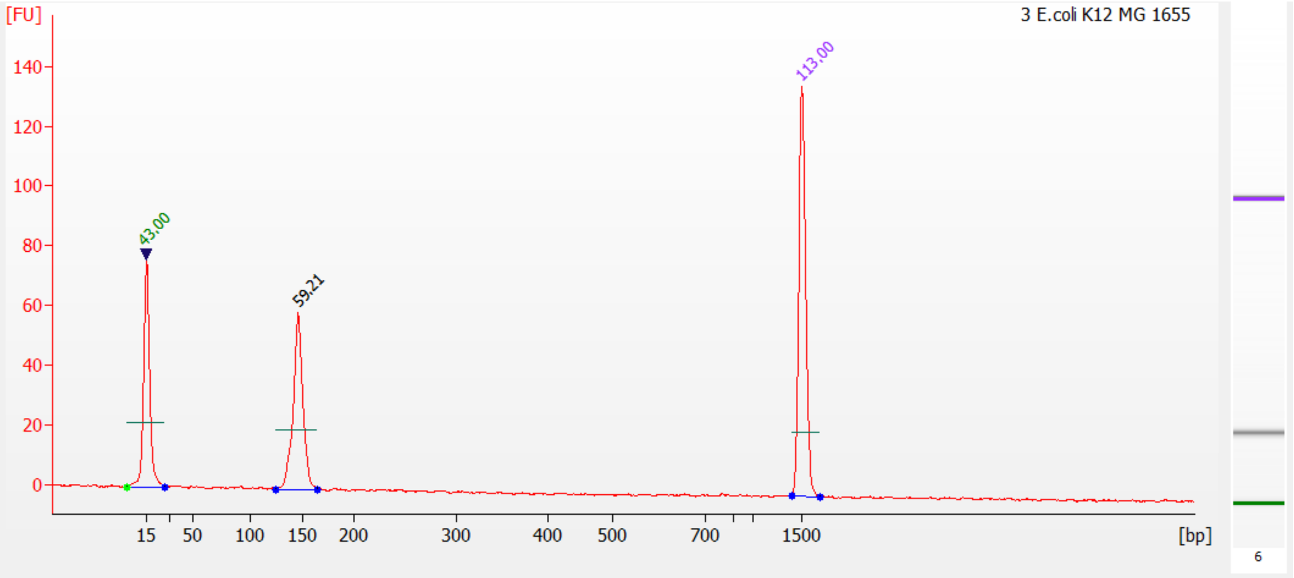

		Size [bp]	Conc. [ng/µl]	Molarity [nmol/l]	Observations
▶	◀	15	4.20	424.2	Lower Marker
2		146	2.85	29.6	
3	▶	1,500	2.10	2.1	Upper Marker

Sample ~~4. E. coli K12 MG1655:~~

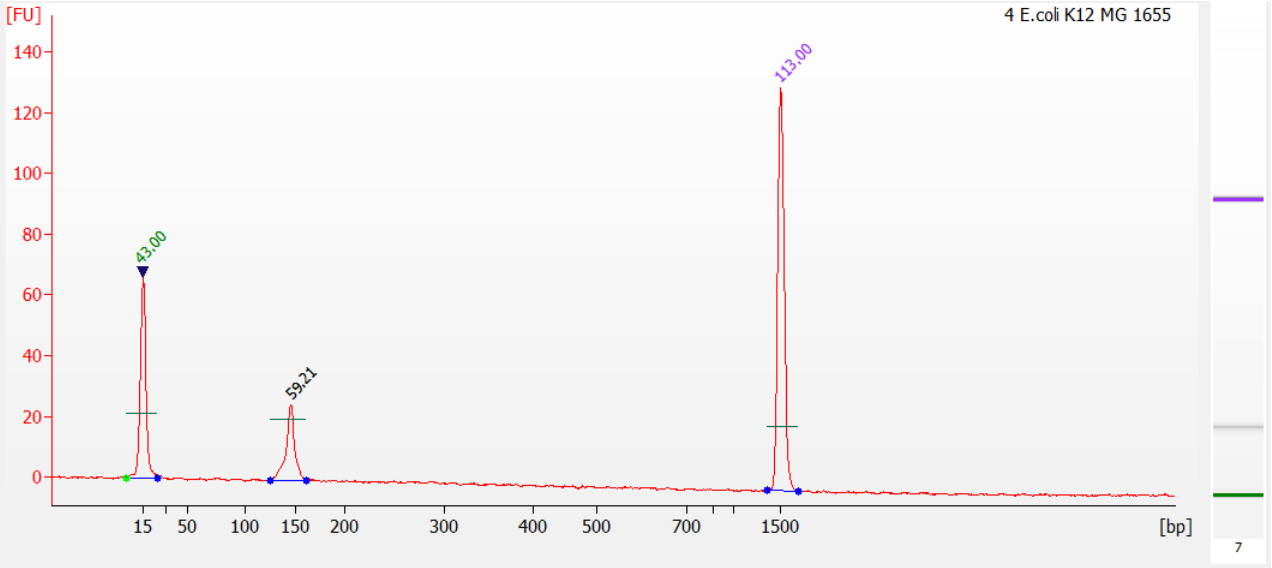

		Size [bp]	Conc. [ng/µl]	Molarity [nmol/l]	Observations
▶	◀	15	4.20	424.2	Lower Marker
2		146	1.16	12.1	
3	▶	1,500	2.10	2.1	Upper Marker

Sample 5. E. coli K12 MG1655:

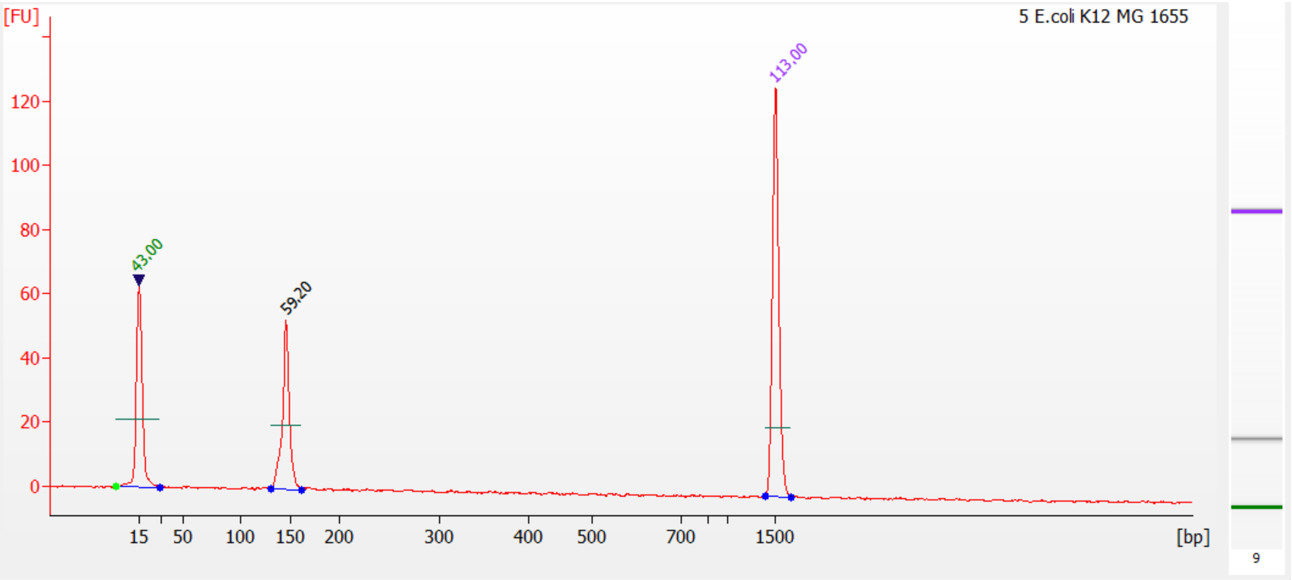

		Size [bp]	Conc. [ng/µl]	Molarity [nmol/l]	Observations
		15	4.20	424.2	Lower Marker
2		146	2.06	21.4	
3		1,500	2.10	2.1	Upper Marker

Sample 7. P. aeruginosa PAO1:

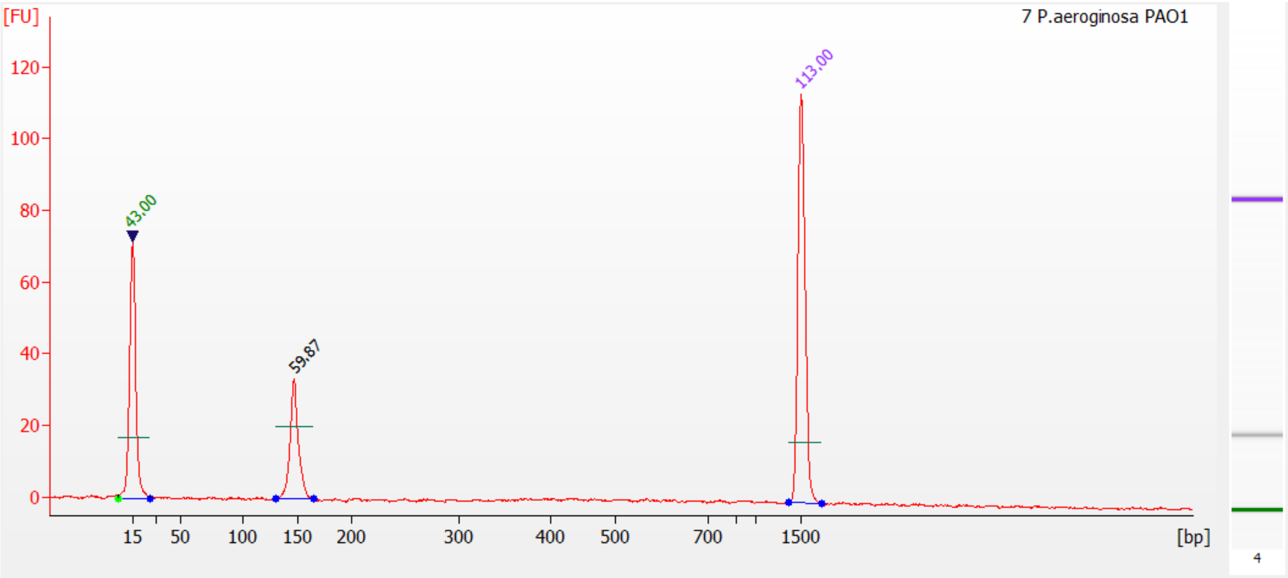

		Size [bp]	Conc. [ng/µl]	Molarity [nmol/l]	Observations
▶	◀	15	4.20	424.2	Lower Marker
2		146	1.59	16.4	
3	▶	1,500	2.10	2.1	Upper Marker

Sample 8. P. aeruginosa PA7:

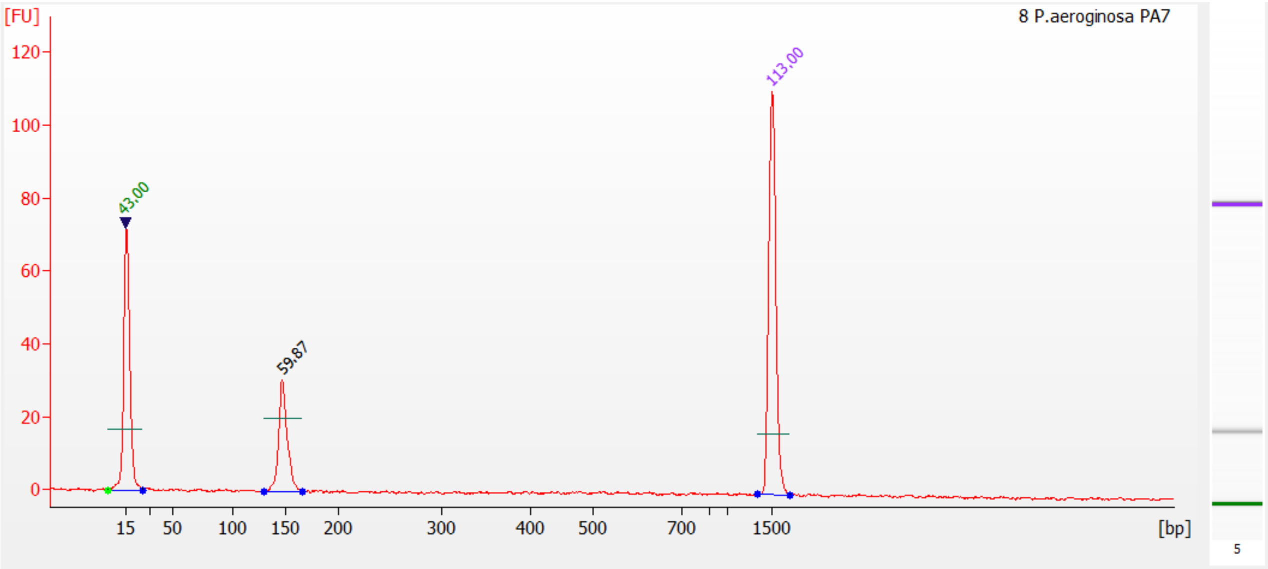

		Size [bp]	Conc. [ng/µl]	Molarity [nmol/l]	Observations
▶	◀	15	4.20	424.2	Lower Marker
2		146	1.57	16.3	
3	▶	1,500	2.10	2.1	Upper Marker

Sample 9. S. aureus:

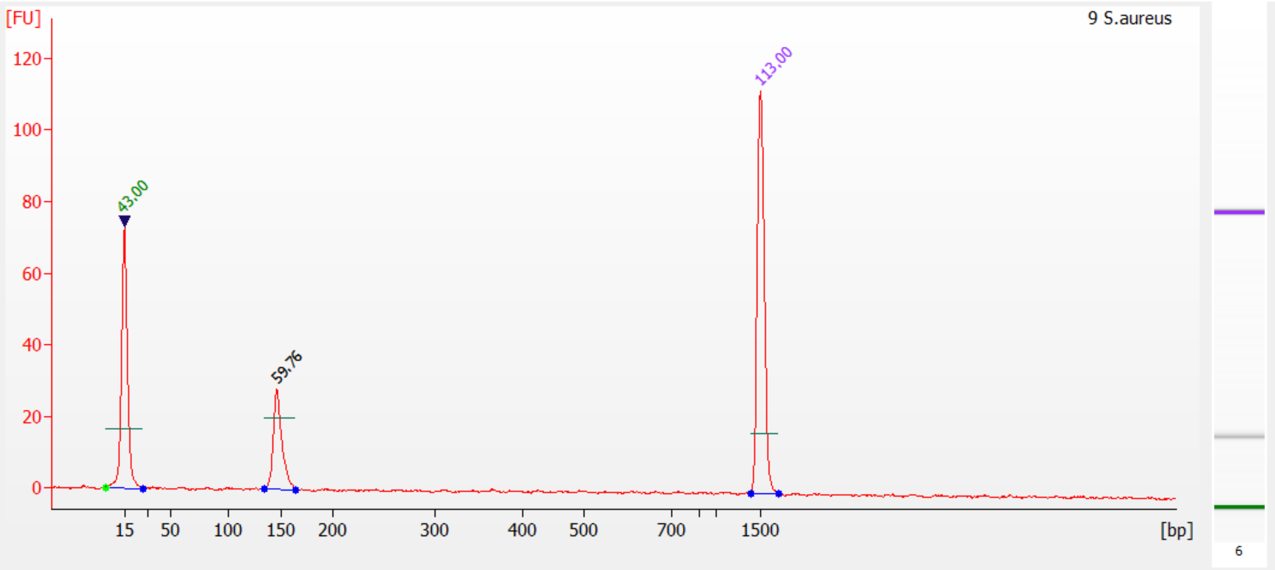

		Size [bp]	Conc. [ng/µl]	Molarity [nmol/l]	Observations
▸	◀	15	4.20	424.2	Lower Marker
2		145	1.40	14.6	
3	▶	1,500	2.10	2.1	Upper Marker

Sample 10. L. pneumophila

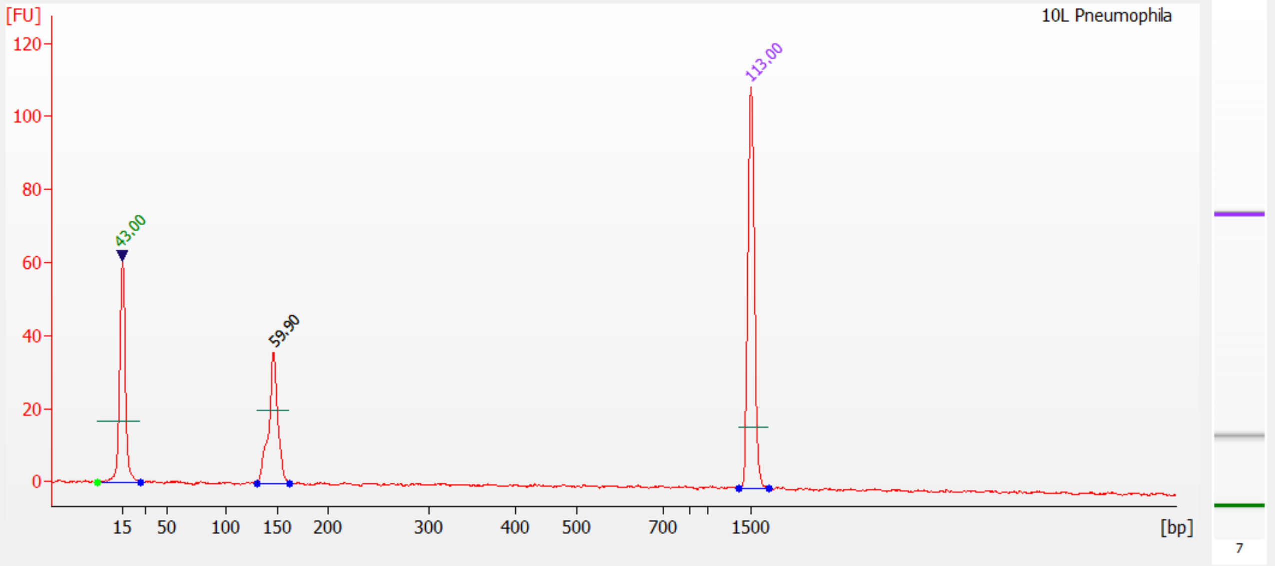

		Size [bp]	Conc. [ng/µl]	Molarity [nmol/l]	Observations
▸	◀	15	4.20	424.2	Lower Marker
2		147	2.16	22.3	
3	▶	1,500	2.10	2.1	Upper Marker

Sample 11. S. thyphimurium:

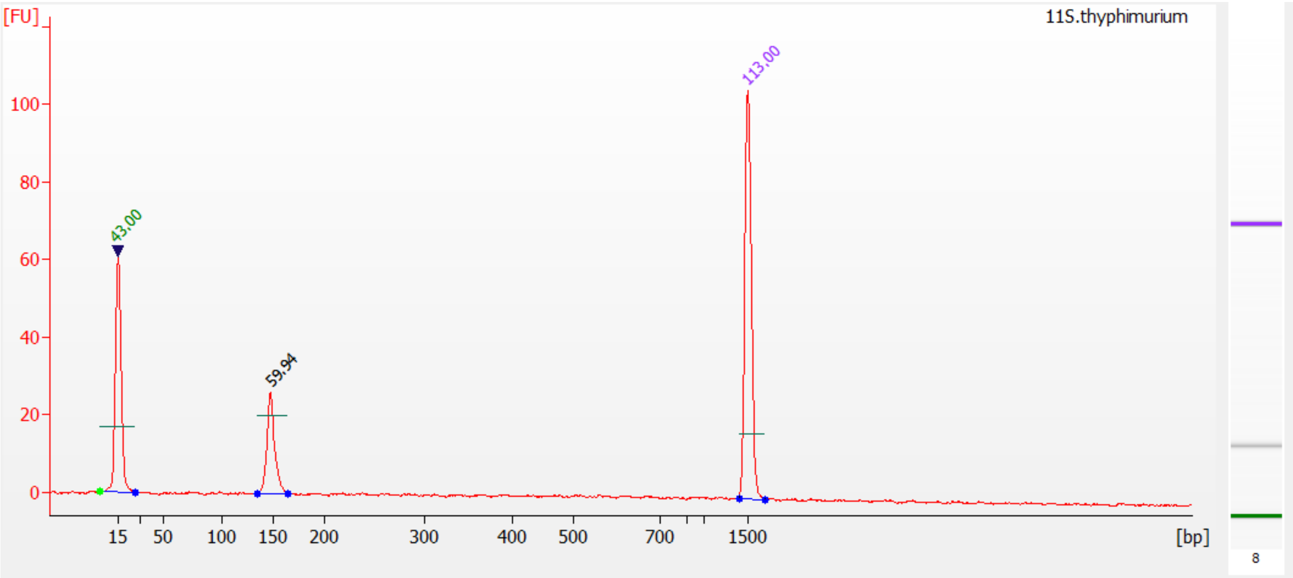

		Size [bp]	Conc. [ng/µl]	Molarity [nmol/l]	Observations
▶	◀	15	4.20	424.2	Lower Marker
2		147	1.34	13.8	
3	▶	1,500	2.10	2.1	Upper Marker

Sample 12. RNA E. coli OMV

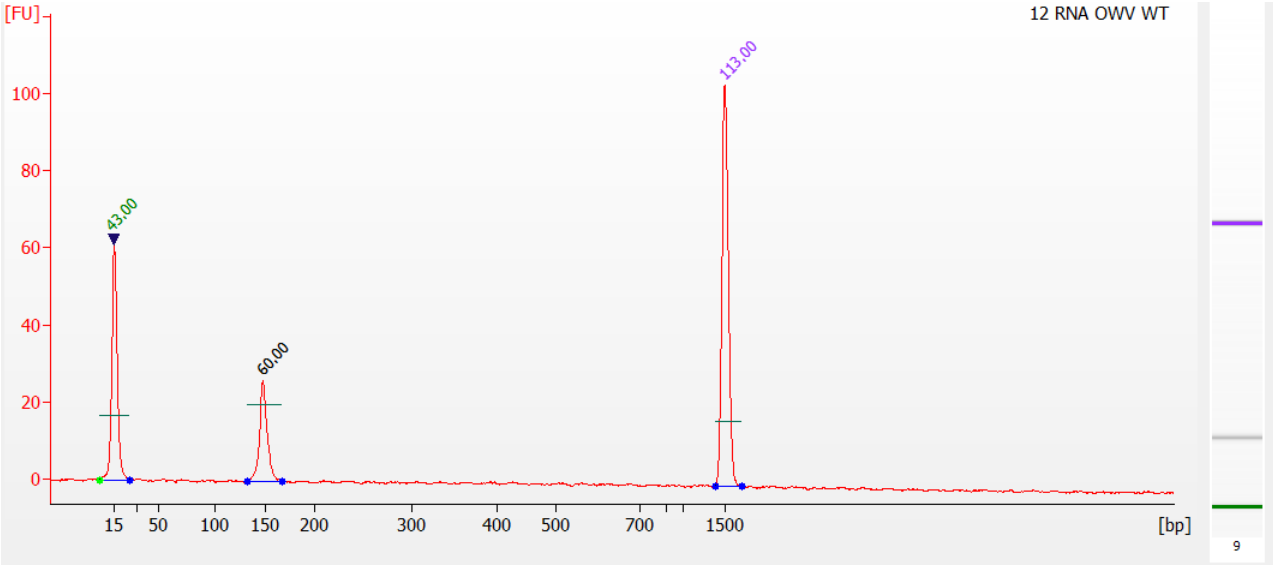

		Size [bp]	Conc. [ng/µl]	Molarity [nmol/l]	Observations
▶	◀	15	4.20	424.2	Lower Marker
2		147	1.40	14.4	
3	▶	1,500	2.10	2.1	Upper Marker

Sample 14. LB Culture medium:

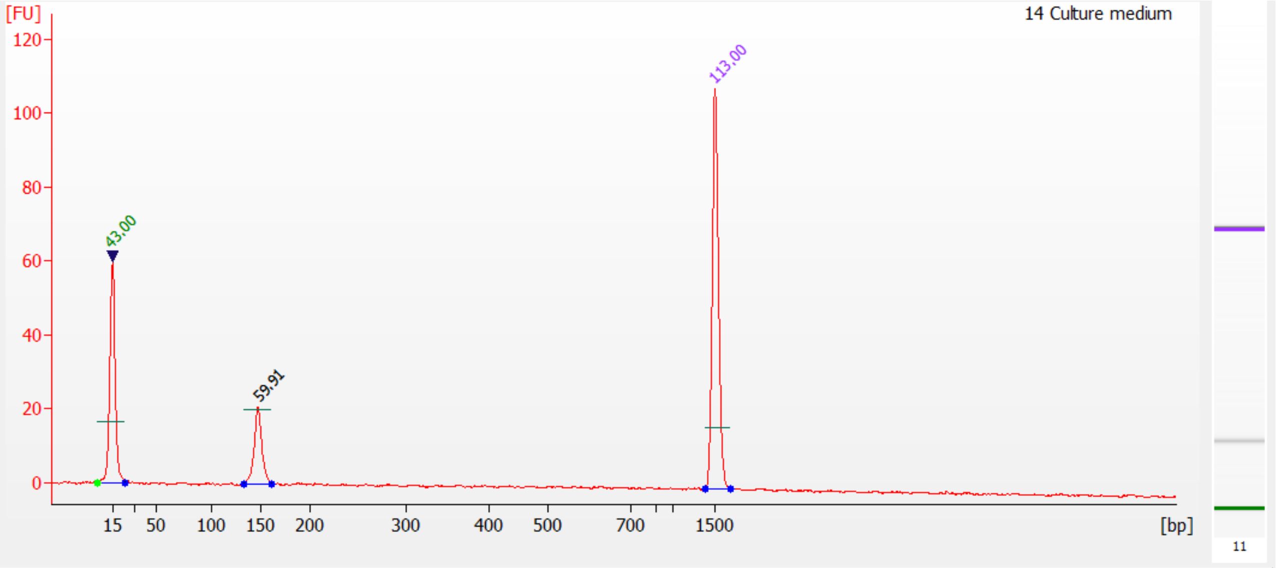

		Size [bp]	Conc. [ng/µl]	Molarity [nmol/l]	Observations
		15	4.20	424.2	Lower Marker
2		147	1.09	11.2	
3		1,500	2.10	2.1	Upper Marker

Arraystar, Inc. 9430 Key West Avenue Suite 128 Tel: 240-314-0388 Email: support@arraystar.com
Rockville, Maryland 20850. USA Fax: 240-238-9860 Website: www.arraystar.com
