# Supplementary material for: RNA Sequencing Unveils Very Small RNAs With Potential Regulatory Functions in Bacteria
Source: Front Mol Biosci. 2022 Jun 3;9:914991. doi: 10.3389/fmolb.2022.914991 (PMC9203972; doi:10.3389/fmolb.2022.914991)
Supplement: Supplementary file 2 [file DataSheet2.docx]

Original research manuscript submitted for publication to *Frontiers in Molecular Biosciences*

**RNA sequencing unveils very small RNAs with potential regulatory functions in bacteria**

Idrissa Diallo^1^, Jeffrey Ho^1^, David Lalaouna^2,§^, Eric Massé^2^ and Patrick Provost^1*^

^1^ CHU de Québec-Université Laval Research Center/CHUL Pavilion, 2705 Blvd Laurier, Quebec City, QC, G1V 4G2, Canada, Department of Microbiology, Infectious Diseases and Immunology, Faculty of Medicine, Université Laval, Quebec City, QC, G1V 0A6, Canada.

^2^ CRCHUS, RNA Group, Department of Biochemistry and Functional Genomics, Faculty of Medicine and Health Sciences, Université de Sherbrooke, 3201 Jean Mignault Street, Sherbrooke, QC J1E 4K8, Canada

§ Current affiliation : Université de Strasbourg, CNRS, ARN UPR 9002, F-67000 Strasbourg, France

* Corresponding author: Dr. Patrick Provost

CHU de Québec-Université Laval Research Center/CHUL Pavilion

2705 Blvd Laurier, Room T1-65

Quebec, QC, G1V 4G2, Canada

Phone: 1 418 525 4444 (ext. 48842)

E-mail : [patrick.provost@crchudequebec.ulaval.ca](mailto:patrick.provost@crchudequebec.ulaval.ca)

**Keywords: RNA sequencing, bacteria, *E. coli*, outer membrane vesicle (OMV), very small RNA (vsRNA), tRNA fragment (tRF**)

**SUPPLEMENTARY FIGURES AND TABLES**

**Table S1. List of bacterial strains and species used in the study.**

| N* | Bacteria | Background | Media | Growth phase | Inoculum | OD_600nm_ | Incubation | Conditions | Stress |
| --- | --- | --- | --- | --- | --- | --- | --- | --- | --- |
| 1 | *E. coli* | K12 - MG1655 | LB | Exponential | ON culture  (1/1000 in 50mL) | 0.443 | 2h45 | Growth at 37°C | Reference |
| 2 | *Salmonella* Typhimurium | WT 14028S (MPBs30) |  |  |  | 0.430 | 3h20 |  |  |
| 3 | *Staphylococcus aureus* | WT HG001  (AEs1) | BHI |  | ON culture  (1/100 in 50mL) | 3.18 | 3h20 |  |  |
| 4 | *Legionella pneumophila* | WT JR32 Philadelphia-1  (LPDL5) | AYE |  | ON culture  (1/50 in 50mL) | 2.99 | 13h |  |  |
| 5 | *Pseudomonas aeruginosa* | PAO1  (T3SS+; exlA-) | LB |  | ON culture  (1/100 in 30mL) | 0.412 | 4h05 |  |  |
| 6 |  | PA7  (T3SS-; exlA+) |  |  |  | 0.433 | 4h15 |  |  |

**Displayed above in details, are the backgrounds and growth conditions of the bacteria. ON = overnight culture**

**Table S2. Adapter-trimmed reads length distribution in 6 bacterial strains.**

| nt | *E. coli* | *L. pneumophila* | PA7 | PAO1 | *S.* Typhimurium | *S. aureus* | *E. coli* OMVs |
| --- | --- | --- | --- | --- | --- | --- | --- |
| 8 | 380611 | 57524 | 5107 | 7807 | 368298 | 119259 | 17999 |
| 9 | 418353 | 98113 | 8611 | 12466 | 409297 | 225813 | 42625 |
| 10 | 593110 | 130236 | 22402 | 32075 | 587177 | 349576 | 89022 |
| 11 | 555462 | 162654 | 37932 | 52350 | 619093 | 442945 | 156317 |
| 12 | 509988 | 203108 | 73587 | 93853 | 533244 | 521071 | 237869 |
| 13 | 716571 | 295676 | 147024 | 212254 | 599014 | 1044038 | 647556 |
| 14 | 579275 | 261458 | 193146 | 222102 | 577952 | 471588 | 575978 |
| 15 | 511777 | 320358 | 234316 | 226518 | 489640 | 456585 | 502156 |
| 16 | 522805 | 435704 | 280995 | 267157 | 580171 | 453577 | 618784 |
| 17 | 496606 | 374697 | 292531 | 274528 | 510119 | 417693 | 457843 |
| 18 | 529119 | 368219 | 313372 | 293295 | 467527 | 412040 | 571558 |
| 19 | 452323 | 312020 | 330752 | 312979 | 393066 | 463663 | 439988 |
| 20 | 373813 | 303486 | 330138 | 313614 | 296501 | 384335 | 278228 |
| 21 | 359818 | 308817 | 328486 | 316075 | 276238 | 289201 | 228228 |
| 22 | 305373 | 300837 | 333748 | 314033 | 278035 | 315721 | 218866 |
| 23 | 268119 | 282218 | 333010 | 310617 | 211198 | 233563 | 248407 |
| 24 | 250691 | 250490 | 356500 | 341647 | 214201 | 206883 | 271218 |
| 25 | 242746 | 232538 | 334801 | 326203 | 186499 | 208179 | 383208 |
| 26 | 265039 | 226903 | 328314 | 314669 | 215777 | 177091 | 182887 |
| 27 | 239395 | 212403 | 312529 | 296201 | 185078 | 145323 | 183898 |
| 28 | 309008 | 217891 | 303856 | 291575 | 268199 | 133766 | 312127 |
| 29 | 800654 | 223082 | 279998 | 279341 | 600247 | 185940 | 300910 |
| 30 | 278751 | 260177 | 300951 | 1646640 | 207012 | 109669 | 320144 |
| Total | **9959407** | **5838609** | **5482106** | **6757999** | **9073583** | **7767519** | **7285816** |
| % RNA_(8-15nt)_ | 43 | 26 | 13 | 13 | 46 | 47 | 31 |
| % RNA_(16-30nt)_ | 57 | 74 | 87 | 87 | 54 | 53 | 69 |

The total numbers of the reads (cleaned after 3’ adapter trimming and passed Solexa CHASTITY quality filter) at the sequencing data processing stages are listed for each of the sequence sizes between 8 and 30nt and for each bacterial sample (*E. coli* MG1655 and its derived OMVs, *L. pneumophila, P. aeruginosa* PA7*, P. aeruginosa* PAO1*, S.* Typhimurium *and S. aureus*). For each sample, the percentage of RNA_8-15nt_ and RNA_16-30nt_ are indicated.

Table S3. Top20 of the most abundant reads in *E. coli* DH5α sample.

| RANK | Reads | Sequences | Possible origins |
| --- | --- | --- | --- |
| 1 | 267567 | AGGCTTGTAGCTC | tRNA-Ile |
| 2 | 61706 | AGGCTTGTAGCTT | tRNA-Ile |
| 3 | 33366 | TGTGGGCACTCGA | 5’ end ETS 16S-23S |
| 4 | 30516 | GGGGCTATAGCTC | tRNA-Ala |
| 5 | 25598 | AGGCTTGTAGCTA | tRNA-Ile |
| 6 | 16704 | AGGGGCGTAGTTC | tRNA-Trp |
| 7 | 8126 | TAAATTGAAGAGT | rRNA 16S |
| 8 | 7205 | AAATTGAAGAGT | rRNA 16S |
| 9 | 5758 | GGGGCTATAGCTT | tRNA-Ala |
| 10 | 5608 | TTTAAATTGAAGAGT | rRNA 16S |
| 11 | 5142 | CGGCACGTAGCGC | tRNA-Pro |
| 12 | 3772 | AGGCTTGTAGCCC | tRNA-Ile |
| 13 | 3399 | TGTGGGCACTCGG | 5’end ETS 16S-23S |
| 14 | 3306 | AGGCTTGTAGCTG | tRNA-Ile |
| 15 | 3159 | TTAAATTGAAGAGT | rRNA 16S |
| 16 | 2422 | GATTGTCTGAT | ITS rRNA 16S -23S |
| 17 | 2397 | GGGGCTATAGCTA | tRNA-Ala |
| 18 | 2235 | AGGGGCGTAGTTT | tRNA-Trp |
| 19 | 2094 | GGGGCTATAGTTC | mRNA fragment |
| 20 | 1893 | CTGTGAGCTCGATG | rRNA 23S |


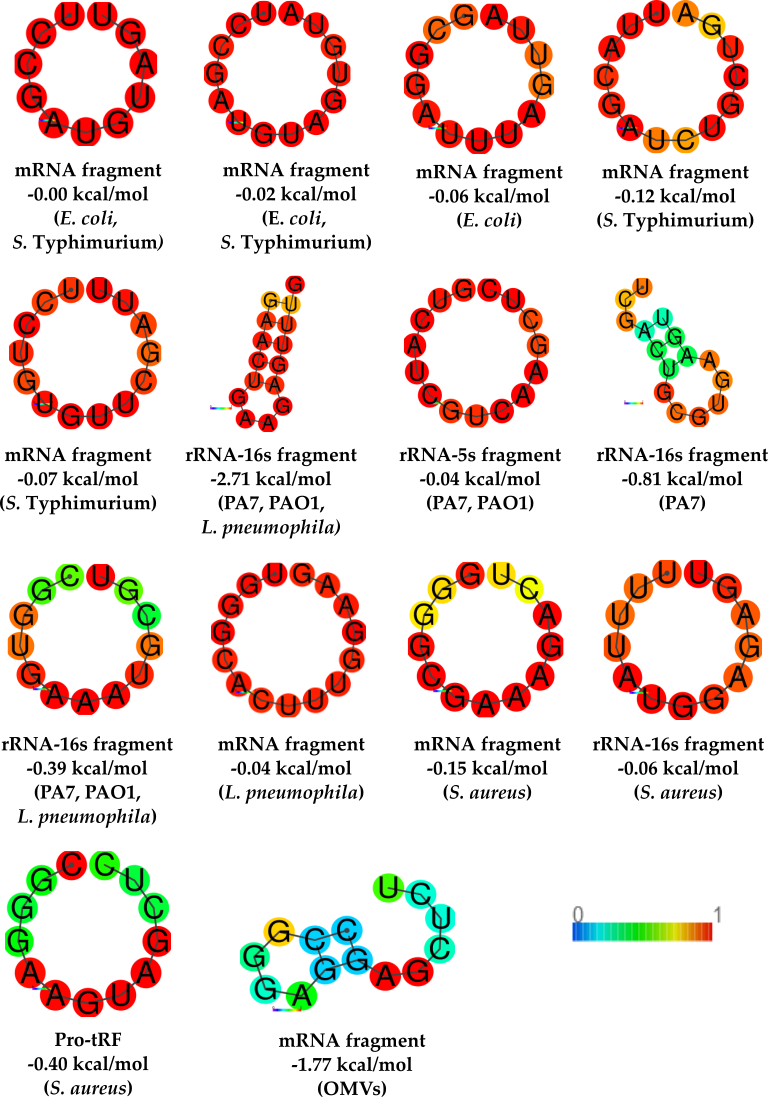
**Figure S1. Representation of the secondary structure of the 6 most abundant tRFs**. RNAfold was used for RNA secondary structure prediction. The values in Kcal/mol represent the minimum free energies. The probability of the base pairs is color-coded 0 to 1, with the red color corresponding to higher confidence.

Table S4. Analysis of reads showing the number of reads found in LB culture medium and those found in *E. coli* sample before and after eliminating LB-mapped sequences.

| **Analysis of sequence reads** | | | |
| --- | --- | --- | --- |
| **Sample names** | **Percentage of reads mapped to media controls (reads)** | **Percentage of reads mapped to the media controls and the *E. coli* genome (no. of reads)** | **Percentage of reads mapped to the *E. coli* genome after removing the reads mapped to the media controls (no. of reads)** |
| RNA*_e. coli_*_-f_ | 41.1 (1751053) | 19.8 (842491) | 34.2 (861282) |
| RNA_OMV-f_ | 41.3 (936952) | 5.23 (118643) | 23 (1332570) |
